# Fishing for subsistence constitutes a livelihood safety net for populations dependent on aquatic foods around the world

**DOI:** 10.1038/s43016-023-00844-4

**Published:** 2023-09-25

**Authors:** John Virdin, Xavier Basurto, Gianluigi Nico, Sarah Harper, Maria del Mar Mancha-Cisneros, Stefania Vannuccini, Molly Ahern, Christopher M. Anderson, Simon Funge-Smith, Nicolas L. Gutierrez, David J. Mills, Nicole Franz

**Affiliations:** 1 Duke Marine Lab, Nicholas School of the Environment, Duke University, Beaufort, NC USA; 2 World Bank, Rome, Italy; 3 Fisheries and Aquaculture Division, Food and Agriculture Organization of the United Nations, Rome, Italy; 4 School of Environmental Studies, University of Victoria, Victoria, British Columbia Canada; 5 Scripps Institute of Oceanography, University of California San Diego, San Diego, CA USA; 6 School of Aquatic and Fishery Sciences and Center for Sustaining Seafood, University of Washington, Seattle, WA USA; 7 FAO Regional Office for Asia and the Pacific, Food and Agriculture Organization of the United Nations, Bangkok, Thailand; 8 WorldFish and Centre for Sustainable Tropical Fisheries and Aquaculture, James Cook University, Townsville, Queensland Australia

**Keywords:** Environmental studies, Sustainability

## Abstract

Fishing for subsistence constitutes a livelihood safety net for poverty, malnutrition and gender inequality for populations dependent upon aquatic foods around the world. Here we provide global estimates showing that almost the same amount of small-scale fishers engage in subsistence fishing at some point during the year as in commercial employment and use subsistence estimates to measure small-scale fisheries’ livelihood safety net function. In 2016, we estimate that 52.8 million people were engaged in subsistence fishing at some point during the year, while another 60.2 million people were commercially employed (90% of global fisheries employment). From 14 country case studies, it was possible to estimate that the subsistence catch provided an average apparent intake of six nutrients critical for positive health outcomes, equivalent to 26% of the recommended daily nutrient intake for 112.5 million people, higher than the national average contribution of beef or poultry.

## Abstract (web summary)

Data from 78 countries and 14 case studies, disaggregated by employment, subsistence and gender along the aquatic food supply chain, are used to estimate the number of livelihoods supported by small-scale fisheries.

## Main

Though they receive less attention from policymakers than other primary economic activities such as agriculture^1^, the world’s small-scale fisheries support livelihoods by providing both monetary income and subsistence food, functioning as critical safety nets that allow people worldwide to avoid poverty and malnutrition. This safety net function is highly relevant for marginalized households and local economies coping with the effects of a changing climate^2^. Yet, characterizing and quantifying the livelihood safety net role of small-scale fisheries has been elusive and often ambiguous in the literature. The lack of information also contributes to inattention from policymakers^3^. The goal of this paper is to help fill this void by using national household survey datasets to estimate the global number and types of small-scale fisheries livelihoods, complemented by national-level case studies.

Within the scholarship on the role of small-scale fisheries in supporting livelihoods and alleviating global poverty, two models have dominated the discussion: a ‘welfare model’ emphasizing *prevention* of poverty^4–6^ and a ‘wealth-based model’ focused more on poverty *reduction*^7^. Case studies supporting the welfare model focus on the role of small-scale fisheries in frequently absorbing surplus labour (that is, unemployed) and playing a safety net function in the context of high vulnerability, thought to be one of the most important contributions these fisheries make to poverty alleviation^5,8–10^. In other case studies, small-scale fisheries operate through wealth-based mechanisms to alleviate poverty through increased labour productivity growth to raise household incomes, increasing community-level economic diversity and resilience to shocks^7^. The multi-dimensional nature of poverty is more complex than these two polar models convey, and aspects of both mechanisms are observed empirically^9,11^. However, because the two models envision opposing policy interventions—wealth-based approaches favour access and harvest controls while welfare-based approaches focus on need-based harvest and participation—understanding the contexts in which each mechanism is more effective in alleviating poverty has important policy implications.

To understand the importance of small-scale fisheries in poverty alleviation and the type of mechanism in use in a given context, it is critical to reliably estimate the number of people dependent upon these fisheries for livelihoods^5^. Over the last decade, governments worldwide have improved the methodology and coverage of national labour force and household surveys that better account for seasonal differences in participation in livelihood-supporting activities. However, these data have been underutilized in building a more complete picture of the global safety net function of small-scale fisheries. The surveys distinguish employment from subsistence fishing activity, allowing us to provide global estimates of the extent of these different livelihood strategies within small-scale fisheries worldwide, disaggregated by demographic and geographic characteristics.

Analysing surveys from 78 countries, we estimated the total number of livelihoods supported by small-scale fisheries worldwide, measured as small-scale fisheries workers sub-divided as either working for employment or subsistence (Methods include definitions of these mutually exclusive terms) and capturing the full range of participants throughout the supply chain, disaggregated by gender. This analysis was supplemented by estimates from 14 case studies of the magnitude of the nutrition safety net provided by subsistence fishing, calculated by applying modelled nutrient profiles for fish to estimates of per capita fish availability from subsistence catch. To identify the extent of the livelihood safety net function of small-scale fisheries, we define that function broadly to include: (1) serving as a buffer against crises for vulnerable households, (2) absorbing excess labour, (3) supporting occupational multiplicity and/or (4) providing food production alternatives. We use subsistence fishing activity as a coarse quantitative proxy for the minimum global extent of this safety net function of small-scale fisheries (that is, the number of people for whom small-scale fisheries provide livelihood safety nets), as it meets this broad definition. While the safety net function of small-scale fisheries is certainly not limited to subsistence fishing activity, the extent and frequency of this activity worldwide, linked with the apparent fish consumption and the estimated nutritional value derived from this consumption, can provide a coarse quantitative proxy indicator of the minimum extent to which small-scale fisheries function as a safety net to help alleviate poverty and malnutrition, within the context of total livelihoods supported by these fisheries. This lower bound definition excludes cases where employment in small-scale fisheries may function as a livelihood safety net; available data typically do not make this distinction, thus the total safety net contribution of small-scale fisheries is bounded between identifiable subsistence activity in small-scale fisheries and total activity in small-scale fisheries.

Using these definitions and through application of existing datasets from national household surveys, we have provided global and regional measures of the number of livelihoods supported by small-scale fisheries and have distinguished the types of livelihood supported: either via employment or subsistence. Doing so provides global and regional gender-specific measures of the number of subsistence fishers, filling a gap from previous efforts (for example, ref. ^12^) and serves as a coarse proxy indicator for the minimum extent to which small-scale fisheries worldwide provide a safety net function to alleviate poverty.

## Results

### Total number of small-scale fisheries livelihoods

**Fig. 1:**
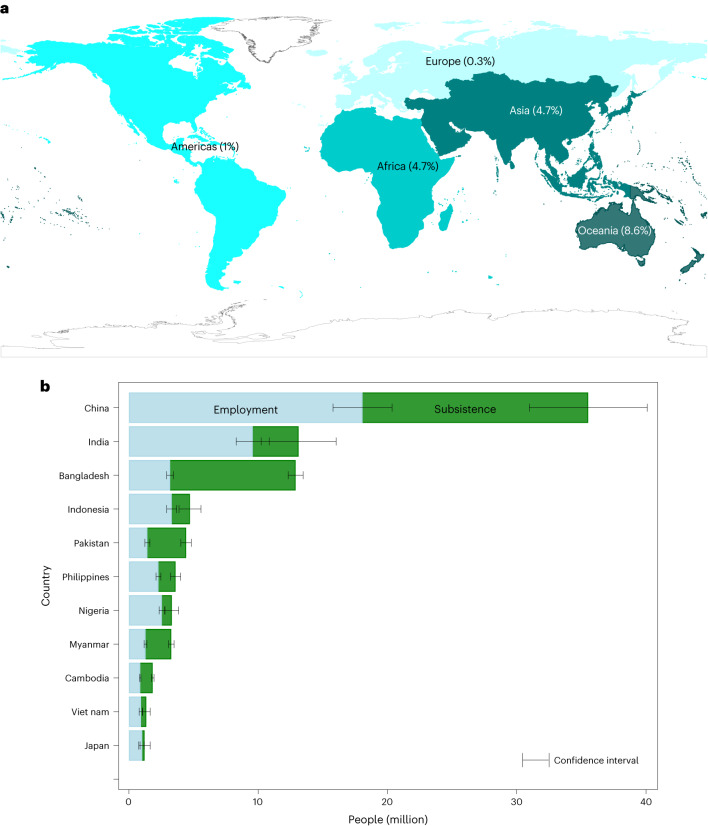
Geographic distribution of small-scale fisheries workersʼ employment and subsistence fishing activity for 2016. **a**, Distribution of people engaged in small-scale fisheries employment and subsistence fishing activity as a proportion of total employment, by region. **b**, Ten countries with the most small-scale fisheries employment and those engaged in subsistence fishing activity. Data are presented as mean values ± (1.96 × (standard error of the mean)) (*N* = 186). Data extrapolated from household-based surveys for 78 countries. Credit: basemap in **b**, UNGIS, UNGSC, Field Missions (https://www.un.org/geospatial). Source data

Our findings show that within small-scale fisheries in 2016, almost the same amount of fishers engaged in subsistence fishing (52.8 million people) as in commercial employment (60.2 million people, accounting for 90% of all small and large-scale fisheries employment worldwide, supporting previous estimates^12–14^. The global estimates for subsistence were not available before. Asia was home to most of the world’s small-scale fish workers, with slightly more people (46.5 million) engaged in small-scale fisheries in 2016 for subsistence fishing activity than for employment (46.1 million) (Fig. 1a). Asia is also home to nine out of the top ten countries ranked by number of small-scale fish workers (Fig. 1b).

At the national level, just ten countries in Africa and Asia are home to an estimated 73% of all people employed part or full time in small-scale fisheries, and China alone is home to 29% (and approximately one-third of all subsistence workers worldwide) (Supplementary Table 1). However, comparison to total employment can indicate the global importance of some countries’ small-scale fisheries but does not capture their relatively high contribution to total livelihoods in some smaller countries, for example, two out of every ten employed workers in many small Pacific Island countries. In terms of small-scale fisheries employment as a percentage of total employment, five of the top ten countries in the world are Pacific Island countries (from 186 countries or territories with data available; Supplementary Table 2), and 108 countries or territories have at least one person employed in small-scale fisheries for every 100 persons employed in all sectors.

### Total number of people engaged in subsistence fishing

**Fig. 2:**
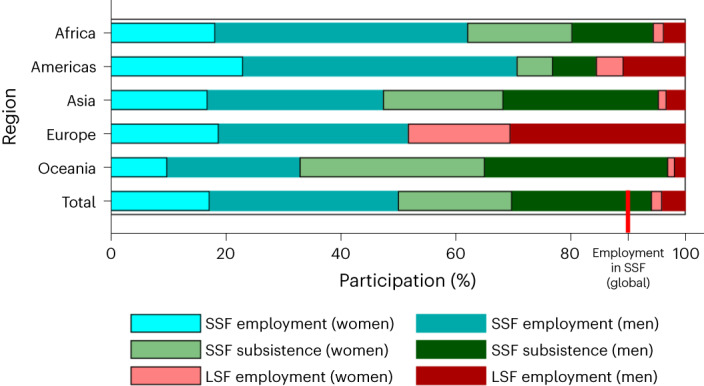
Estimated participation in fisheries employment and subsistence activity by gender and region for 2016. The red vertical line captures the share of small-scale fisheries (SSF) employment in total employment in fisheries. Table 1 provides 95% confidence intervals. Data extrapolated from household-based surveys for 78 countries. LSF, large-scale fisheries. Source data

The number of people engaging in small-scale fisheries primarily for subsistence provides an entry to quantify the livelihood safety net function that these fisheries play globally. The 52.8 million people estimated to engage in subsistence fishing activity at least once per year (Table 1) represents 47% of all workers in small-scale fisheries (113.1 million) for 2016. Approximately 60% of those estimated to engage in subsistence fishing activity are found in low or lower-middle-income countries and 29% are found in ‘low-income food-deficit countries’ (LIFDCs), with an additional 33% in China. Within LIFDCs, 62% of the people estimated to work in small-scale fisheries do so for subsistence only, though this ratio drops to 48% if Bangladesh is excluded. In Asia and Oceania, the majority of the people estimated to work in small-scale fisheries do so for subsistence only (just over 50% and 66% respectively), although for countries where data is available, they generate only a fraction of the total volume of catch from small-scale fisheries^15^. In Africa, the Americas and Europe, the majority of people working in small-scale fisheries do so commercially (employed), though in Africa over a third of small-scale fisheries workers do so for subsistence only (Fig. 2). Globally, for both inland and marine fisheries, more people participate in fisheries for subsistence than for commercial harvest and processing combined (but not trading) (Table 1). The majority (68%) of subsistence fishing activity occurred in inland fisheries, though more people engaged in marine subsistence fishing activity than for commercial harvesting (Table 1).

**Table 1:** Regional and global estimates of the number of workers in small- and large-scale fisheries in 2016 (millions) at each stage of production

	Subsistence fishing activity			Commercial fishing employment (part and full time)						
				Pre-harvest	Harvest		Post-harvest		Total employment	Total (subsistence + employment) inland and marine
				Inland and marine	Inland	Marine	Processing	Trading		
Region	Inland	Marine	Total subsistence							
**Small-scale fisheries**										
Africa	3.45	1.21	**4.66** *(3.33*–*5.97)*	0.31	2.65	1.36	1.37	3.28	**8.97** *(7.86*–*10.06)*	**13.63**
Asia	31.65	14.80	**46.45** (*38.76*–*53.89)*	1.01	11.25	9.80	5.24	18.84	**46.14** *(40.47*–*51.80)*	**92.59**
Europe	ND	ND	ND	0.16	0.06	0.17	0.18	0.42	**0.99** (0.65–1.31)	**0.99**
Oceania	0.69	0.34	**1.03** *(0.91*–*1.14)*	0.02	0.11	0.18	0.08	0.14	**0.53** *(0.48*–*0.58)*	**1.56**
The Americas	0.21	0.49	**0.70** *(0.27*–*0.99)*	0.23	0.53	1.36	0.63	0.85	**3.60** *(2.87*–*4.33)*	**4.30**
**Total**	**36.0**	**16.84**	**52.84** *(43.27*–*61.98)*	**1.73**	**14.60**	**12.87**	**7.50**	**23.53**	**60.23** *(52.33*–*68.07)*	**113.07**
**Large-scale fisheries**										
Africa	NA	NA	NA	0.02	0.02	0.19	0.30	0.30	**0.83** *(0.55*–*1.09)*	**0.83**
Asia	NA	NA	NA	0.24	0.68	1.16	0.85	1.80	**4.73** *(3.00*–*6.44)*	**4.73**
Europe	NA	NA	NA	0.15	0.01	0.19	0.28	0.29	**0.92** *(0.60*–*1.22)*	**0.92**
Oceania	NA	NA	NA	0	0	0.01	0.02	0.02	**0.05** *(0.04*–*0.07)*	**0.05**
The Americas	NA	NA	NA	0.05	0.01	0.19	0.28	0.27	**0.80** *(0.49*–*1.10)*	**0.80**
**Total**	NA	NA	NA	**0.46**	**0.72**	**1.74**	**1.73**	**2.68**	**7.33** *(4.68*–*9.91)*	**7.33**
**Total capture fisheries**										
Africa	3.45	1.21	**4.66**	0.33	2.67	1.55	1.67	3.58	**9.80**	**14.46**
Asia	31.65	14.80	**46.45**	1.25	11.93	10.96	6.09	20.64	**50.87**	**97.32**
Europe	0	0	0	0.31	0.07	0.36	0.46	0.71	**1.91**	**1.91**
Oceania	0.69	0.34	**1.03**	0.02	0.11	0.19	0.10	0.16	**0.58**	**1.61**
The Americas	0.21	0.49	**0.70**	0.28	0.54	1.55	0.91	1.12	**4.40**	**5.10**
**Total**	**36.0**	**16.84**	**52.84**	**2.19**	**15.32**	**14.61**	**9.23**	**26.21**	**67.56**	**120.40**

Note: figures in italics represent the upper and lower bounds at 95% confidence level; figures in bold represent sums; NA, not applicable; ND, no data.

Though the extent of women’s work in small-scale fisheries has often been overlooked^16^, women account for 45% of people engaged in subsistence fishing activity (and 40% of those employed part or full time in small-scale fisheries or engaged in subsistence), with considerable variation by region (6–32%, excluding Europe) (Fig. 2). For example, participation in subsistence fishing activity, which may also include post-harvest processing such as smoking or drying fish, is much higher in Africa at 57% than for other regions.

### Small-scale fisheries livelihoods found in contexts of high vulnerability

**Fig. 3:**
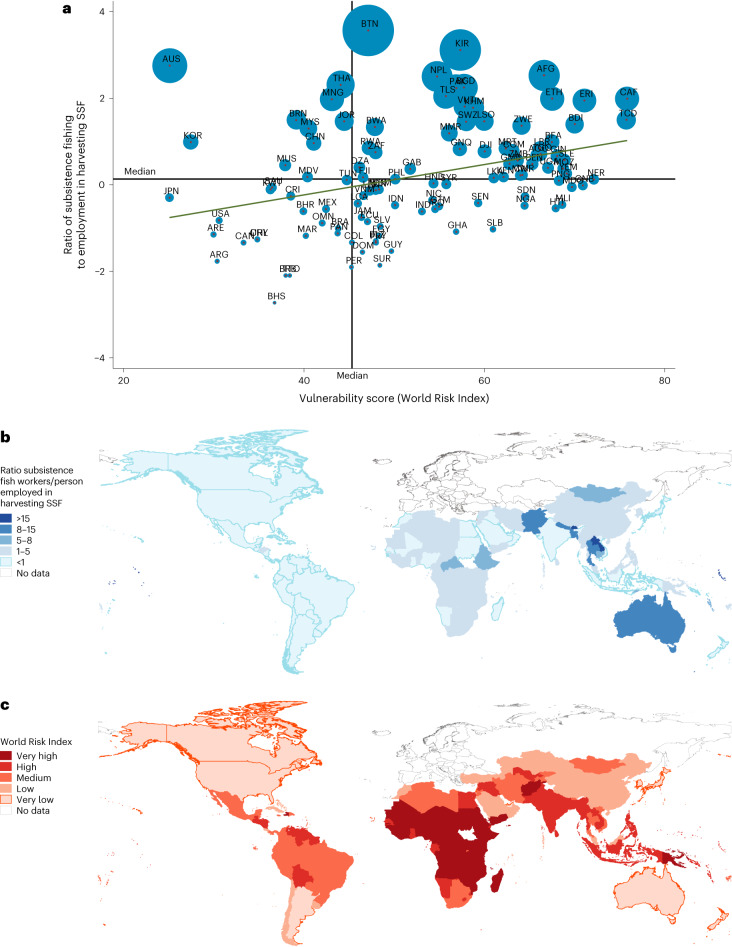
Global distribution of subsistence activity within small-scale fisheries for 2016 and countries with ‘very high’ or ‘high’ vulnerability scores from the World Risk Index for 2021. **a**, Correlation between the ratio of subsistence fish workers in a country and the country’s vulnerability score on the World Risk Index. Dots are scaled to overall size of fishery engagement in each country. **b**, Distribution of concentrations of subsistence fish workers in the harvesting segment of small-scale fisheries. **c**, Degree of national vulnerability to climate according to the World Risk Index. Data for subsistence fish workers and persons employed in harvesting SSF extrapolated from household-based surveys for 78 countries (see Source Data Fig. 3 for full names of abbreviated countries). Credit: basemaps in **b**,**c**, UNGIS, UNGSC, Field Missions (https://www.un.org/geospatial). Source data

One aspect of the safety net function of small-scale fisheries livelihoods is to serve as a buffer against shocks for vulnerable households. While we do not measure vulnerability at the household level, the geographic areas where small-scale fishing livelihoods (employment and subsistence) are most concentrated (lower- to middle-income countries in the tropics) overlap with regions of high human vulnerability to climate change. These regions are characterized by the International Panel on Climate Change as ‘global hotspots of high human vulnerability’ to climate hazards (east Africa, central Africa and west Africa, followed by central America, south Asia and southeast Asia). As one of several global indices of country-level vulnerability, the World Risk Index was created to measure countries’ risk and vulnerability to climatic and natural hazards (based on indicators of hazard and exposure, vulnerability and lack of coping capacity)^17,18^. For the 165 countries and territories with both estimates of small-scale fisheries employment and vulnerability scores on the World Risk Index, 54% of employment is located in countries ranked as having ‘high’ or ‘very high’ vulnerability, rising to 79% if China is excluded^18^. For the 127 countries and territories with both estimates of subsistence fishing activity and vulnerability scores on the World Risk Index, this share rises slightly, with 57% of subsistence workers located in countries ranked as having ‘high’ or ‘very high’ vulnerability, increasing to 87% if China is excluded. For these countries, the ratio of subsistence workers to employment in the harvesting segment of small-scale fisheries shows a positive correlation with a country’s vulnerability based on the World Risk Index vulnerability score (*r* = 0.345) (Fig. 3a), as many of the countries with higher concentrations of subsistence workers in small-scale fisheries are located in the tropics, with ‘high’ or ‘very high’ vulnerability scores (with exceptions, such as Australia, based on high estimates of subsistence to commercial workers in small-scale fisheries) (Fig. 3b,c).

### Subsistence fishing, excess labour and occupational multiplicity

As an indication of the safety net function of small-scale fisheries, within LIFDCs, the number of people engaged in subsistence fishing activity as a percentage of agricultural employment (including fisheries) is inversely correlated (*r* = −0.52) with the headcount poverty ratio (at US$1.90 per day) (Supplementary Fig. 2). This suggests that although subsistence fishing activity is more likely to be found in lower- and lower-middle-income countries (and China), in countries such as many LIFDCs where this activity is most prevalent as a percentage of agricultural employment, it may help agriculture workers buffer against shocks and supplement access to food and nutrition.

**Fig. 4:**
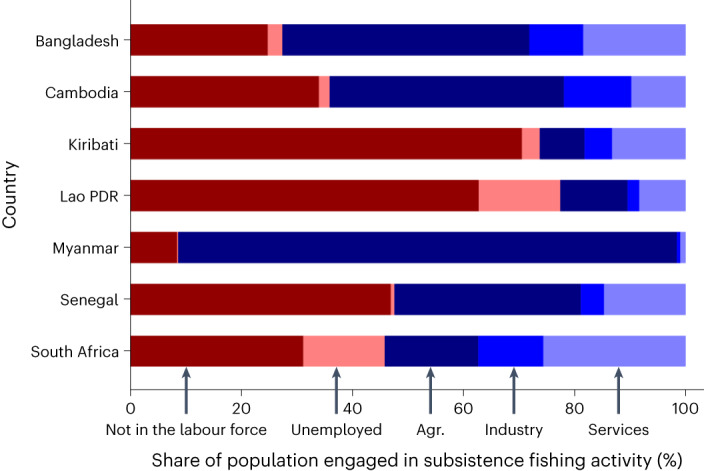
Share of the population engaging in subsistence fishing activity, by main economic sector and labour force status based on data from seven countries. These examples cover different years based on data availability from household-based surveys: Kiribati, 2015; Lao PDR, 2017; Senegal, 2012; Cambodia, 2013; South Africa, 2017; Bangladesh 2013; Myanmar, 2019. Persons engaged in subsistence fishing activity were classified according to their economic sector of employment and labour force status (Methods provide more detail). Source data

While national household survey data only supported global estimates of people working in subsistence fishing activity at a frequency of at least once during the year, in seven countries (representing 23% of the global total of people working in subsistence fishing activity), the data provide more detail, showing that on average, subsistence workers spent 4.2 hours per week on this activity. While subsistence workers depended upon a range of other economic activities in these seven countries, agriculture (crops and livestock) was most prevalent, employing more than 33% of the workers in four of the countries and 90% in Myanmar. On average across the seven countries, 40% of the subsistence fish workers were outside of the formal labour force entirely. Such findings illustrate the variable rates at which these fisheries absorb excess labour and support occupational pluralism (for example, by filling livelihood gaps for agricultural workers) at any given time during the year or during the farming off season (Fig. 4)^5,8–10^. The livelihood safety net role is particularly evident with inland fisheries, where the majority of subsistence fishing activity occurs in Lao PDR (100%), Bangladesh (91%), Cambodia (79%), South Africa (60%) and Myanmar (52%). Lao People’s Democratic Republic (PDR) was the most extreme example globally, where in 2017, 97% of the people working in small-scale fisheries did so only for subsistence (or 17% of the country’s population) (Supplementary Fig. 4) and in the province of Savannakhet alone, 25% of the provincial population (more than 252,000 people) was estimated to engage in subsistence fishing activities, of whom only 8% were employed (that is, persons of working age that are ‘at work’ or ‘not at work’ due to temporary absence).

### Case studies on subsistence fishing as a nutritional safety net

**Fig. 5:**
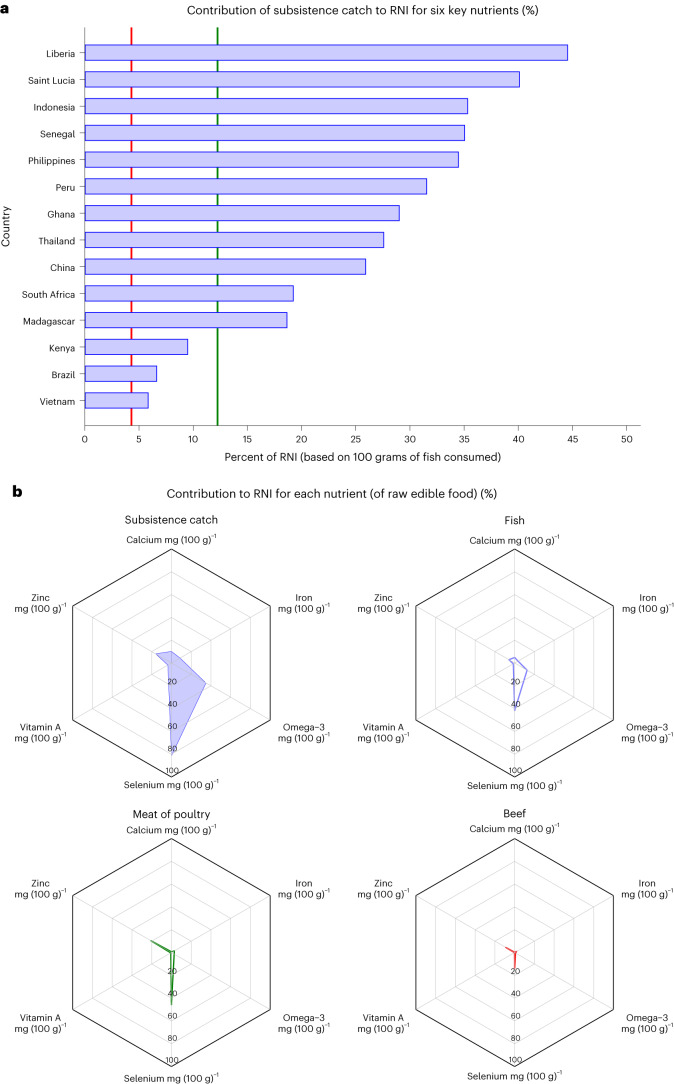
Average contribution of marine and inland subsistence catches to the daily RNI for six nutrients in 14 country case studies. **a**, Mean of six nutrients, calculated by averaging the percent contribution to the RNI of the six nutrients presented in **b**. The vertical lines represent the average percent contribution of the six nutrients to the RNI across the entire population of the same 14 countries for: (1) beef (red line) and (2) poultry (green line). **b**, Average contribution of marine and inland subsistence catches to the daily RNI for six nutrients across the 14 countries analysed (Brazil, China, Ghana, Indonesia, Kenya, Liberia, Madagascar, Peru, Philippines, Saint Lucia, Senegal, South Africa, Thailand and Vietnam), as derived from the nutrient values of all species that form the aggregate volume of subsistence catch in each country, as compared to the average contribution from total small-scale fisheries catch (fish), meat and poultry to the national population in the same 14 countries. Note that the percentage nutrient composition of the subsistence catch and total small-scale fisheries catch is similar; these only appear different in **b** because they are distributed over different population sizes (subsistence fish catch being smaller). The portion size for fish, beef and poultry (that is, apparent per capita consumption) was derived by considering the total population in the 14 countries under study. In contrast, the portion size for subsistence catch was determined solely based on the population engaged in subsistence fishing. Across these 14 countries, the estimated average apparent per capita consumption of subsistence catch is 74.4 grams per day. The apparent per capita consumption of fish, poultry and beef is estimated at 35.7, 51.5 and 15.5 grams, respectively. Source data

The catch from subsistence fishing activity provides a source of nutrients critical for health^15^, functioning as a nutritional safety net in some instances that may support food sovereignty for coastal and riparian communities. Detailed case studies from the Illuminating Hidden Harvests study provided data on subsistence catch for 14 countries (Brazil, China, Ghana, Indonesia Kenya, Liberia, Madagascar, Peru, Philippines, Saint Lucia, Senegal, South Africa, Thailand and Vietnam) for the period 2013 through 2017. The estimated annual average subsistence catch for these countries was 3.3 million tonnes in total for this period (2013–2017) (ref. ^15^), equivalent to 9% of the average annual total catch reported from these countries over the same period^19^. While not globally representative, these 14 countries are important in global fisheries: with the subsistence catch representing 4% of the average annual global catch during this period^19^. This subsistence catch is predicted to have contributed an apparent daily supply for fishers and their households (a total of 112.5 million people) equivalent on average to 26% of their daily recommended nutrient intakes (RNI) of six key nutrients common in global micronutrient deficiencies driving poor health outcomes: iron, zinc, selenium, calcium, vitamin A and omega-3 fatty acids, based on daily consumption of 100 grams of fish (Fig. 5b and Supplementary Table 13). For comparison, for the households supported by the subsistence catch (112.5 million people), the contribution of the six key nutrients exceeded the average contribution from the total small-scale fisheries catch (12%), beef (4%) and poultry (12%) to the national population in the same 14 countries (a total of 2.3 billion people), driven primarily by the contribution of selenium and omega-3 to the RNI and with inter-country differences reflecting apparent per capita supply of subsistence catch (Fig. 5a).

## Discussion

Approximately 44% of all people who participate in fisheries do so for subsistence, and when trading is excluded (because this activity does not occur in subsistence activity by definition), more people work in fisheries for subsistence than for employment. These results illustrate the minimum extent of the livelihood safety net role small-scale fisheries play in riparian and coastal environments around the world (Table 1), often in contexts of high human vulnerability to climate change. Of course, small-scale fisheries are diverse^3^ and in some cases can generate significant economic returns and may even offer opportunities for labour productivity growth within resource constraints^20^. Our analysis has focused only on a global measure of their role as a safety net for households often in vulnerable contexts (using subsistence fishing as a proxy for this role), as a welfare-based mechanism to alleviate poverty, while similar studies could consider household income levels and changes in small-scale fisheries, for example, to better measure the role of the wealth-based mechanism.

From analysis of this one mechanism, the picture that emerges of small-scale fisheries worldwide suggests that they play an important role in poverty alleviation as a safety net (‘welfare model’)—that is, backstopping livelihoods and probably preventing or limiting household poverty, often in countries considered to be contexts of high human vulnerability to climate change. This picture is further illustrated in seven countries where the national household surveys provide more detailed data (Fig. 4), showing that in six of the seven countries, at least a quarter of the people engaged in subsistence fishing were outside of the labour force.

From a nutrition security perspective, the worldwide extent of subsistence fishing activity highlights the important role that small-scale fisheries can play in delivering key nutrients in lower-income countries. The 14 country case studies suggest the potentially critical role that such fisheries play in the diets of an estimated 112.5 million people in aggregate, providing access to six key nutrients (26% of recommended daily intake). In many coastal and riparian areas throughout lower-income countries, this access may help communities maintain varying degrees of nutrition security. Conversely, loss of access to these fisheries could further erode an important nutritional safety net for these households. While subsistence fishing provides an indication of the contributions that small-scale fisheries livelihoods can provide to nutrition, such contributions are not limited to subsistence, as recent examples in Malawi, Uganda and Tanzania highlighted—where households located near small-scale fisheries were 13% more likely to achieve adequate food security and 15% less likely to be income poor than the most distant households^21^.

In conclusion, at a crucial time of climate and economic instability^22^, understanding drivers of livelihood choices in different contexts remains important for policymakers. This study provides a global measure of the minimum extent of the livelihood safety net function of small-scale fisheries, underscoring the key role these fisheries will continue to play in alleviating poverty and malnutrition (particularly in Africa and Asia where almost three-quarters of those employed in small-scale fisheries are found). The significant estimates of the role of small-scale fisheries as livelihood safety nets presented here suggest that policymakers should focus more effort in identifying the distinctive roles, functions and needs of these fisheries in specific coastal and inland areas, and particularly in identifying subsistence fishing. This would shape choices in policy interventions between wealth-based approaches favouring access and harvest controls and/or welfare-based approaches focused on need-based harvest and participation. In contexts where small-scale fisheries do function predominantly as livelihood safety nets, policymakers should focus interventions to help address the vulnerability of communities as a prerequisite to measures addressing resource overexploitation.

## Methods

### Approach and concepts used to characterize the roles that small-scale fisheries play in supporting livelihoods as safety nets

To evaluate the strategies engaged by individuals and households to reduce food and nutrition insecurity and alleviate poverty through small-scale fishery livelihoods—a term used here to refer to the capabilities, assets and activities required for a means of living or adequate stocks and flows of food and cash to meet basic needs^23,24^—two separate measures based on labour statistics were assessed: (1) employment, defined as all persons of working age who, during a short reference period (typically the week before the interview), were engaged in any activity to produce goods or services provided for pay or profit, including both part- and full-time employment to capture seasonal variation (with interviews conducted continuously throughout the year in almost all surveys, including both employed persons ‘at work’, that is, who worked in a job for at least one hour during the reference period, and employed persons ‘not at work’ due to temporary absence from a job or to working-time arrangements, such as shifts in work, flexitime and compensatory leave for overtime); and (2) subsistence, defined as working for one’s own consumption: workers who produce goods or services that are predominantly consumed by their own household, with no transaction occurring in the marketplace^25^, potentially including pre- and post-harvest activity in fisheries (and so referred to here as ‘subsistence fishing activity’, inclusive of any pre- and post-harvest activity). With these definitions, the term workers is used here to refer both to people who work in fisheries for pay (employment) and to those who work for their own consumption (subsistence), consistent with definitions from scholars of subsistence as sustaining a basic level of livelihood^26^. Note that these definitions may lead to underestimations of subsistence fishing activity, as many who we classify as employed because they sell a majority of their product may keep some of the catch for their own consumption.

Following Béné^5^, the safety net function of small-scale fisheries has typically been defined as the role they play in providing food production alternatives (that is, subsistence) and additional income opportunities for vulnerable households with low savings, as needed in times of individual or collective hardship, crisis or stress (for example, to cope with a climate-related shock or reduced agricultural production). For this study we broaden the definition of safety net to include the separate but similar poverty alleviation functions of absorbing excess unskilled labour and adding to the portfolio of livelihood options for vulnerable households. While the function has typically been defined more narrowly to focus on buffering vulnerable households in times of crisis^5^, parsing between these functions is unrealistic even at the household level and ultimately unnecessary as all contribute to the role of small-scale fisheries in preventing poverty (welfare mechanism).

As for other primary economic activities such as agriculture, a significant portion of the employment and subsistence in small-scale fisheries is irregular and seasonal, located in rural areas and conducted in parallel with other non-fisheries activities to provide additional income or nutritional support, particularly in inland fisheries to fill ‘livelihood gaps’ during the agriculture off seasons^27–29^. Acknowledging different usages^26^, by definition, subsistence fishing activity often provides supplementary or alternative food for households in times of shocks or limited employment^30^ or forms part of livelihood strategies including multiple occupations—for example, a majority of subsistence fishing households in coastal South Africa were found to be involved in multiple occupations and food insecure^31^; similarly in Savannaket, Laos, occupational diversity was significantly and positively associated with the probability of fishing, largely for subsistence^32^. While certainly not limited to subsistence fishing activity, the extent and frequency of this activity worldwide, linked with the apparent fish consumption and the estimated nutritional value derived from this consumption, can provide a coarse quantitative proxy indicator of the minimum use of small-scale fisheries as a safety net to help alleviate poverty and malnutrition within the context of total livelihoods supported by these fisheries.

While we consider the presence and frequency of subsistence fishing activity as a coarse proxy for the minimum use of small-scale fisheries as a livelihood safety net, and acknowledging that not all subsistence fishing activity may serve the function of a safety net, the measure may still understate the importance of this function. Beyond subsistence, many who work in small-scale fisheries for employment may also do so in ways that could be characterized as a safety net, engaging in the activity as part of pluralist strategies or in response to shocks. Similarly, some who are counted as employed because they sell a majority of their product may still keep some of the catch for their own consumption.

### Sources of data

For this study, three different types of large-scale and standardized household survey instrument provided a previously unused source of data on small-scale fisheries livelihoods: population censuses, labour force surveys and household income and expenditure surveys conducted by governments’ national statistics agencies (Supplementary Information and https://www.ilo.org/surveyLib/index.php/home). Survey data from at least one of these instruments were available for 78 countries over the period 2008 to 2018 (with 54 of the 78 surveys conducted in 2014 or subsequently), representing almost 79% of the world population in 2016 (labour force surveys for 33 countries, household income and expenditure surveys employment modules for 44 countries and a PC for one country). Data collected in the surveys were classified according to common standards that allowed for cross-country comparison based on the type of activity undertaken as defined by the International Classification of Economic Activity and the International Standard Classification of Occupations standards. Questions on employment were asked about the activity conducted within the previous week as the reference period, while for subsistence the reference period was typically once within the previous year, but in both cases interviews were conducted throughout the year to reflect seasonal variation and generate annual average participation (additionally, many national surveys in Asia and Latin America collect quarterly data via panel samples or sub-samples interviewed four times during the year). To be representative, the surveys aimed for national coverage with samples randomly selected from a listing of households (‘master sampling frame’) typically created on the basis of the most recent population census and stratified by geography (‘enumeration areas’). These data are generally available to the public upon request from national statistical offices.

### Data collection, organization and gap filling

The questions included in the household surveys provided the data for the analysis. For questions related to employment, respondents were first asked (yes/no) if they are employed (that is, if they ‘did any work for a wage, salary or commission’, ‘ran a family (farm or non-farm) business’, ‘helped in the household (farm or non-farm) business or ‘had a job or business s/he will return to’ for at least one hour during the reference period, typically the previous week), and for those who answer yes, a series of subsequent questions were asked, including their occupation and economic sector of employment. For questions related to subsistence activity, respondents identified as employed were asked ‘are the products obtained from this activity for sale/exchange or for family use’ and were able to select one of the four following options: (1) ‘only for sale/barter’, (2) ‘mainly for sale/barter’, (3) ‘mainly for family use’ or (4) ‘only for family use’ (with responses indicating (3) or (4) categorized here as engaged in subsistence activities).

Responses to these questions were categorized according to the type of activity performed by gender. Those persons engaged in activities related to fisheries were further classified based on their type of work, which allowed for identification of those employed in fisheries (and subsequently in small-scale fisheries) and those engaged in fisheries activities for subsistence, in both cases by gender. In addition to the category specifically labelled for ‘fishing activities’ and related to the harvesting stage, other categories of activity were cross checked and included to identify employment in pre- and post-harvest activities as different stages of the production process (Supplementary Table 5; for example, ‘fish processing’, ‘wholesale of fishery products’ and so on).

#### Identification of employment in small-scale fisheries

In the absence of a universal definition of small-scale fisheries^3^, we follow the practice of the International Classification of Status in Employment^33^ to characterize operations in different sectors as small-scale based on employment classifications. Following this practice, employment in fisheries was classified in the surveys as either paid or self-employed, with the latter sub-divided into ‘employers’, ‘own-account workers’ and ‘contributing family workers’. To disaggregate this employment between small- and large-scale fisheries, those persons classified as ‘own-account workers’ and ‘contributing family workers’ were assumed to participate in small-scale fisheries. Of the remainder, those persons employed in enterprises whose total number of workers was less than two-thirds of the 90th percentile number of workers engaged in all fisheries-related enterprises within a given country were assumed to participate in small-scale fisheries. This same operational criteria—based on the context-dependent threshold and the status in employment—was also applied to those who engage in pre- and post-harvest activities connected to fisheries as a proxy for operations linked to small-scale harvesting, acknowledging that in some cases, large enterprises may process fish caught by small-scale harvesters and vice versa. The cut-off of two-thirds of 90th percentile was meant to capture the average number of workers employed in the top 10% of the largest business operations in fisheries. The choice of the 90th percentile, rather than a crude absolute threshold that is applied without distinction across countries, can better capture differences in the size of business across countries. In this regard, depending on the country-specific distribution of the number of employees and its corresponding relative average number of workers in the largest 10% of fishing operations, small-scale fishing operations in one country can be classified as large-scale in another country. The average number of workers in the largest 10% of fishing operations (90th percentile) was further adjusted by two-thirds to also include in large-scale fisheries those employees working in the intersection of small- and large-scale fishing business.

#### Identification of persons engaged in subsistence fishing activity

Data on subsistence fishing activity (defined as working for ‘own consumption’, including potentially pre- and post-harvest activity) were available in 32 of the 78 national surveys used, with those engaged in subsistence fishing activity in these countries equivalent to 81% of the total number of people employed in fisheries in the same countries. For the majority of these surveys, data were only available to estimate persons engaged in subsistence fishing activity at a frequency of at least once during the previous year, based on the reference period. For nine surveys (representing 25% of the total number of persons estimated globally to be engaged in subsistence fishing activity), the data permits to estimate subsistence fishing activity at a higher frequency of at least one hour during the previous month, or in some cases during the previous week. Additionally, the surveys in Cambodia and Laos (0.96 million and 1.06 million estimated people participating in subsistence fishing activity respectively) were conducted only once per year but confirmed that the activity occurred within the previous week (that is, the recall period). Finally, the surveys from seven countries (representing 23% of the estimated global total of persons engaged in subsistence fishing activity) provide data showing that on average those engaged in subsistence fishing activity spent 4.2 hours doing so per week reported, suggesting a significant investment of time in the activity (and justifying an assumption that the global total of persons estimated to be engaged in subsistence fishing activity did so more frequently than once per year).

#### Organization of the data and estimates to fill gaps

The microdata from the 78 national household-based surveys were processed, harmonized and reported at the national level for each country. To allow for comparison, the results in the 78 national datasets were adjusted to the study year 2016 (the study year chosen for the Illuminating Hidden Harvests assessment, based on more recent middle year of the period for which data was collected: 2013–2018) by taking the ratio of employment for the survey to the International Labour Organization (ILO) data on the total population employed in agriculture, forestry and fishery in that country and applying it to the ILO data for that population in 2016 (Supplementary Box 3). Where data were missing within the 78 national datasets, they were estimated by calculating and applying ratios from the mean of available data from other countries within the same geographic archetype. Geographic archetypes were specified at the lowest possible regional grouping, according to regional groupings used by the ILO. The most common gaps and the ratios applied to fill them are described in the Supplementary Information and for missing gender-disaggregated data Supplementary Box 4.

### Global extrapolation from the 78 national datasets

The results from the 78 national datasets were extrapolated to the regional level using the geographic archetypes from ILO and subsequently to the global level. To correct for non-response bias in countries not included in the national datasets (which were selected based on the availability of information and not randomly), a weighted regression analysis based on independent variables considered as predictors was used, following recommendations by the ILO^34^. Weights of the different predictor variables were calculated as the inverse probability of selection (or inverse propensity score) to account for differences between the 78 countries for which data were collected and the world’s remaining countries to which the results were extrapolated (Supplementary Table 8). Using these weights to correct for non-response bias, the weighted regression analysis was conducted, essentially generating estimates based on assumed relationships between employment, subsistence and livelihood dependency variables and a set of predictor variables. The predictor variables used were chosen based on (1) strong correlation with the outcome variables (measured by the R squared) (Supplementary Table 9) and (2) availability worldwide. These included (1); Employment in agriculture, forestry and fishery; (2) Employment in industry and employment in services; (3) Total population; (4) gross domestic product (GDP) per capita (purchasing power parity); (5) GDP growth; (6) Value added in agriculture, forestry and fisheries. For marine small-scale fisheries, the additional predictor ‘Length of coastline (km)’ was included, whereas for inland small-scale fisheries, it was ‘Area of inland water bodies’ (Supplementary Table 9 provides predictor variables used).

As a cross check, the resulting estimates were compared to: (1) data compiled on small- and large-scale fisheries employment in 58 national-level case studies and (2) government responses to a survey conducted by the United Nations Food and Agriculture Organization (FAO) with all member states during 2018 and 2019, conducted as part of the Illuminating Hidden Harvests global assessment of small-scale fisheries (Supplementary Fig. 7). Additionally, results were compared to publicly available datasets on aggregated employment in fisheries: (1) the International Labour Organization labor statistics database (ILOSTAT) data on employment in either fishing or aquaculture (aggregated) and (2) FAO data on employment in fisheries (aggregated between small and large-scale fisheries) (Supplementary Fig. 8). For countries where significant differences emerged, experts were consulted to help provide further explanations and eventually adjust estimates from the weighted regression analysis. A final check was comparison to a dataset of the global marine fishing effort, disaggregated between small and large-scale fishing, to identify any countries where zero small- or large-scale fishing effort occurred, but the estimates from surveys suggested a non-zero employment in fish harvesting^35^. Finally, the results are comparable to previous studies, such as World Bank^12^ (Supplementary Information provides more detail on the process of cross checks).

### Selection and use of national datasets for more in-depth case studies on the safety net function of small-scale fisheries

National datasets were used for more in-depth analysis of the safety net function where information was available. In 14 of the datasets (Bangladesh, Brazil, Cambodia, Chile, Egypt, the Gambia, India, Indonesia, Mexico, Peru, Senegal, Sierra Leone, Tunisia and Yemen), sufficient information was available to assess the role of small-scale fisheries employment in total employment at a sub-national level. Additionally, in seven datasets, more detailed information was available on subsistence fishing activity (Fig. 4), including on how these workers allocated their labour (Kiribati, Lao PDR, Senegal, Cambodia, South Africa, Bangladesh and Myanmar). For the ten countries shown in Fig. 3 to have the highest ratio of the number of people engaging in subsistence fishing at some point during the year to the number of people employed part or full time in small-scale fisheries (Lao PDR, Kiribati, Bangladesh, Cambodia, Myanmar, Senegal, Sierra Leone, South Africa, Indonesia, the Gambia), this list was calculated based on national datasets with available observations, rather than the global dataset including estimates. The list of the top ten countries worldwide would change if it included the countries with data based on estimates.

### Estimates of the nutritional safety net function of small-scale fisheries

From the Illuminating Hidden Harvests assessment, data were compiled on a wide range of indicators of the contributions of small-scale fisheries to society, including the volume of catch landed, by species, in 58 country case studies. For each of these country case studies, researchers either compiled available data on the use of the catch (the percentage of the volume landed that was used for commercial sale and destined for human consumption domestically, commercial export, subsistence or for non-human consumption), by species, or used expert judgement to estimate^15^. For 14 of the 58 countries, there were sufficient data on catch use to estimate the average total volume of catch for subsistence by species for the period from 2013 through 2017 (multiplying the volume of catch landed for each species by the percentage reported or estimated by experts to be used for subsistence).

For each of the 14 countries, the estimated volume of subsistence catch was assumed to be divided evenly between the persons engaged in small-scale fisheries and their household members to generate an estimated per capita consumption of subsistence catch by species (mg per day) for a total number of persons. Drawing upon nutrient data compiled from peer-reviewed publications and existing databases for over 500 marine and inland fish species, the Illuminating Hidden Harvests assessment developed a model for predicting the species’ composition of six nutrients commonly deficient and driving poor health outcomes: iron, zinc, selenium, calcium, vitamin A and omega-3 fatty acids^15^. Using this model, the volume of subsistence catch by species in each of the 14 countries was converted into volumes of each of the six nutrients assumed to be consumed by the population of subsistence fishers and their households. A significant volume of the catch in each of the 14 countries was not identified by species (‘not elsewhere included’) (Supplementary Table 11), and the nutrient composition imputed based on the mean nutrient values of species caught by marine or inland small-scale fisheries within the same country (Supplementary Information). Finally, the volume of the six nutrients from the subsistence catch consumed by a given population in each of the 14 countries was compared to the minimum recommended nutrition intake (RNI) for each.

### Statistics and reproducibility

The study was designed as an analysis of existing data from 78 standardized national household-based surveys, conducted by governments’ national statistics agencies from 2008 to 2015. No available surveys and data were excluded from the analysis. To fill in any gaps in data in surveys and to estimate results for any countries without surveys, we used standard econometric modelling (weighted regression analysis). For the case studies, the subsistence catch volume data were used from country case studies conducted in the global Illuminating Hidden Harvests study, and a predictive modelling approach developed for that study was used to predict the nutrient composition of the catch. Data gaps were filled using the mean nutrient values of species belonging to the same country, same sector or detailed functional group.

### Reporting summary

Further information on research design is available in the Nature Portfolio Reporting Summary linked to this article.

### Supplementary information

Supplementary Information Supplementary Figs. 1–14, Tables 1–14 and Discussion.

Reporting Summary

Supplementary Data Data sheets (including statistical source data for Table 1), labelled as: Data source, Employment in SSF, Women employment in SSF, Employment in LSF, Women employment in LSF, Subsistence workers and Subsistence workers women.

### Source data

Source Data Fig. 1 Statistical source data.

Source Data Fig. 2 Statistical source data.

Source Data Fig. 3 Statistical source data.

Source Data Fig. 4 Statistical source data.

Source Data Fig. 5 Statistical source data.

## Data Availability

The results for estimated livelihoods by country and the references for sources are publicly available. The source data from the national household surveys conducted by governments are available from each government’s National Statistics Office, which were used with permission for the current study and are generally publicly available upon request. Data are also available from the authors upon request and with permission of the government’s National Statistics Office. The results presented and sources of data (surveys used) are available in the Supplementary Data file. Source data are provided with this paper.

## Code availability

Data were stored in Excel and Stata software. The analysis was performed in Stata and is available.
