# Supplementary material for: Fishing for subsistence constitutes a livelihood safety net for populations dependent on aquatic foods around the world
Source: Nat Food. 2023 Sep 25;4(10):874–85. doi: 10.1038/s43016-023-00844-4 (PMC10589092; doi:10.1038/s43016-023-00844-4)
Supplement: Supplementary file 1 — Supplementary Figs. 1–14, Tables 1–14 and Discussion. [file 43016_2023_844_MOESM1_ESM.pdf]

nature food

Article https://doi.org/10.1038/s43016-023-00844-4

Fishing for subsistence constitutes a livelihood safety net for populations dependent on aquatic foods around the world

In the format provided by the authors and unedited

Supplementary Materials

I. Additional Information referenced in the Article

Table S1. Ten countries with the largest national estimates of part and full-time small-scale fisheries employment in 2016 (number of persons), with number of subsistence fishers included for reference

	Employment (part and full-time)	Subsistence fishing	Total small-scale fisheries participation
China	18,068,356	17,453,780	35,522,136
India	9,580,693	3,541,877	13,122,570
Indonesia	3,317,355	1,406,037	4,723,392
Bangladesh	3,189,814	9,704,662	12,894,476
Nigeria	2,552,434	765,636	3,318,070
Philippines	2,283761	1,322,176	3,605,937
Pakistan	1,429,764	2,992,800	4,422,564
Myanmar	1,287,058	1,988,939	3,275,997
Japan	1,022986	187,109	1,210,095
Vietnam	930,463	416,875	1,347,338
**Total**	43,662,684	39,779,891	83,442,575

Table S2. Ten countries with the highest estimated portion of employment in small-scale fisheries in 2016 (small-scale fisheries employment per 100 persons employed in all sectors)

Country	Persons employed in small-scale fisheries per 100 persons employed in all sectors*
Tonga	27.5
Samoa	24.0
Solomon Islands	23.4
Cambodia	10.0
French Polynesia	9.99
Mali	9.89
Papua New Guinea	8.97
Maldives	8.23
Senegal	7.86
Saint Vincent and the Grenadines	6.53

**Employment in all sectors from ILOSTAT (Total employment – ILO modelled estimates)*

Figure S2. Correlation between the share of subsistence fishers in agriculture employment (%) and the poverty headcount ratio at $1.90 in low-income food-deficit countries (LIFDCs)

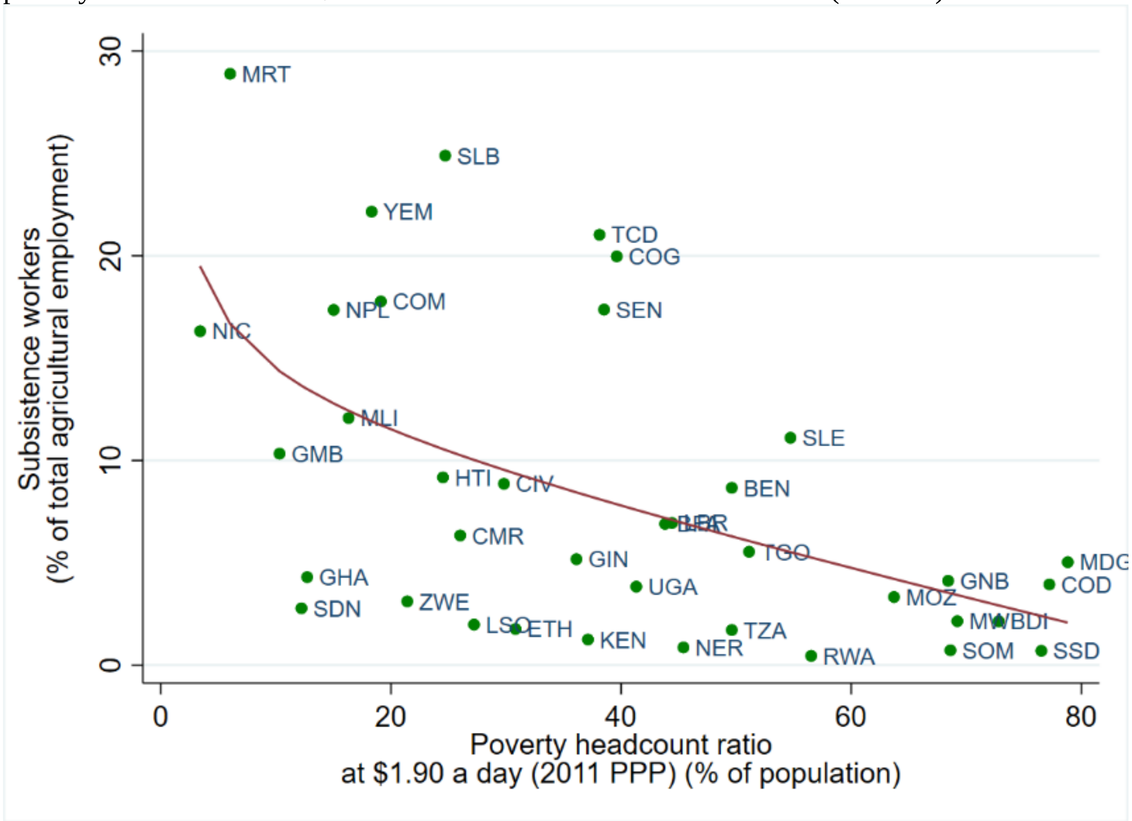

*Source***:** Number of subsistence workers estimated based on the methodology presented in this study: ILOSTAT for total employment in agriculture (https://www.ilo.org/shinyapps/bulkexplorer20/?lang=en); World Bank WDI for the poverty headcount ratio https://data.worldbank.org/ *Note*: total agricultural employment in the denominator of the indicator in the vertical axis includes all employment in primary food production activities, including crops, livestock, forestry, fisheries and aquaculture, according to ILOSTAT.

Figure S3. Mean daily income of persons employed in small-scale fisheries compared to the mean daily income of persons engaged in fisheries for subsistence (a) and cumulative distribution function of the average daily income generated by persons employed in small-scale fisheries and those engaged in subsistence (b), in the three countries of Bangladesh, Cambodia and Laos

Panel a. Panel b.

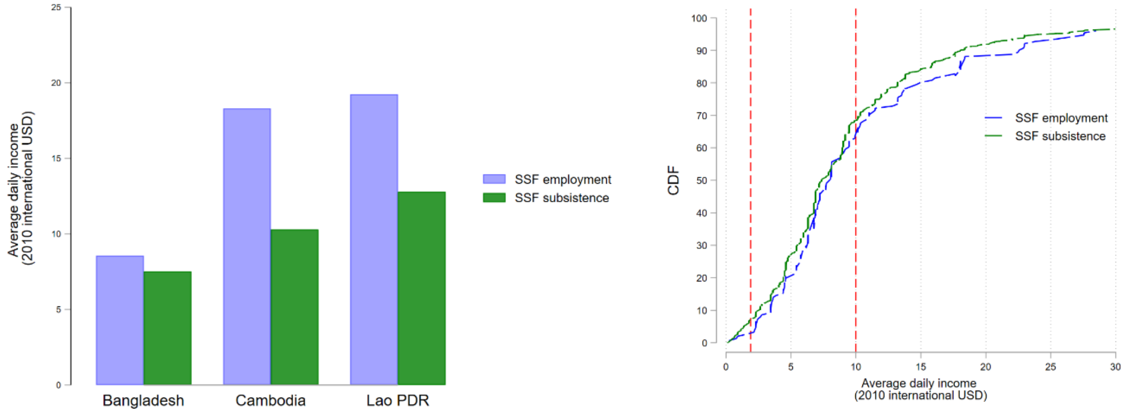

These examples cover different years based on data availability: Bangladesh 2013; Cambodia, 2013; Lao PDR 2017. Note: the mean daily income reported by subsistence workers in small-scale fisheries is generated from employment activities performed outside subsistence fishing.

Panel (a) shows the mean daily income of people who also engage in subsistence fishing compared to the mean daily income of those employed in small-scale fisheries. Panel (b) shows the cumulative distribution functions of the mean daily income for those who engage (blue line) in subsistence fishing and those employed in small-scale fisheries (black line). On the y-axis, each point on the two curves measures the share of workers with a mean daily income equal or lesser than a given reference threshold. The analysis of the CDFs suggests that the distribution of the mean daily income of those who are engaged in subsistence fishing always lies to the left of those who are employed in small-scale fisheries.

This implies that the distribution of the daily income of subsistence workers in small-scale fisheries displays first order stochastic dominance over the distribution of those who are employed in small-scale fisheries. Consistently, for any level of daily income taken as a reference point, the share of the population who earn lesser than the reference threshold is always higher for those who engage in subsistence fishing, compared to those who are employed in small-scale fisheries. For example, in the three countries analyzed the share of subsistence workers in small-scale fisheries whose mean daily income is less than 1.9 international USD per day (i.e. the extreme poverty line) is 3.4 percentage points higher compared to those who are employed in small-scale fisheries, in aggregated. When the threshold is increased up to 10 international USD per day, in the three countries analyzed the share of subsistence workers in small-scale fisheries who earn less than 10 international USD per day is always higher compared to those the corresponding share of those who are employed in small-scale fisheries, respectively +14 percentage points in Cambodia, +9 points in Lao's and +2 points in Bangladesh.

Figure S4. Percentage of persons participating in small-scale fisheries who are subsistence workers* in ten national examples

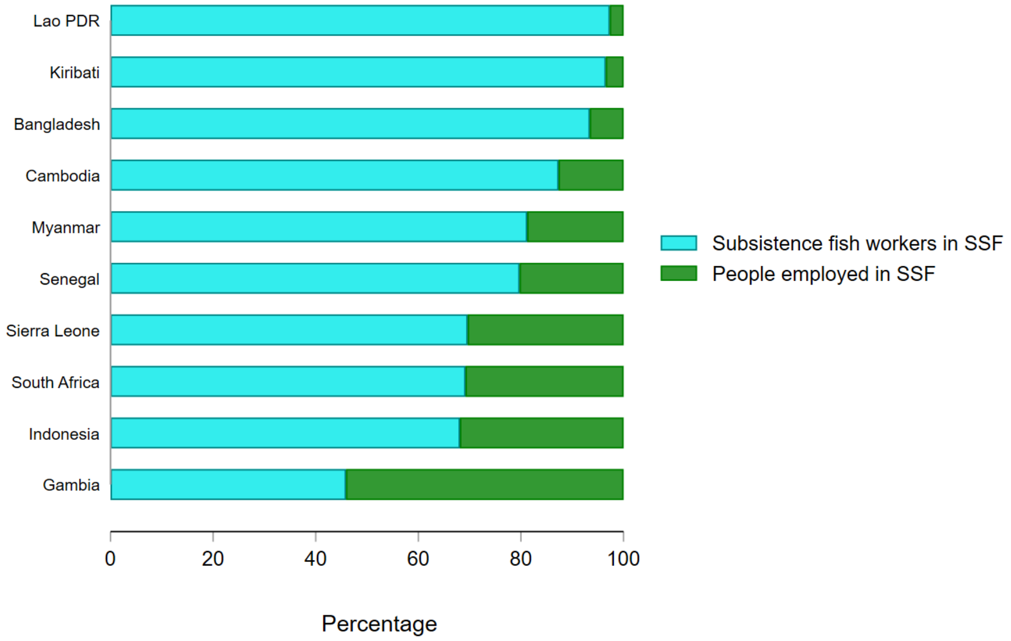

**These examples snapshot different years based on data availability: Lao PDR 2017; Kiribati, 2015; Bangladesh 2013; Cambodia, 2013; Myanmar, 2019; Senegal 2012; Sierra Leone, 2011; South Africa, 2017; Indonesia, 2015; The Gambia, 2016. The blue and red bars measure the distribution of employment and subsistence fishing activity in small-scale fisheries (left-side y-axis). The blue dots measure the ratio of subsistence fishing activity to employment in small-scale fisheries (right-side y-axis). Top ten countries with highest ratio of number of people engaging in*

subsistence fishing at some point during the year to the number of people employed part or full-time in the small-scale fisheries. The list is based on the top ten countries with sufficient data provided in national datasets, rather than datasets with estimates.

In nine of the ten countries with the highest ratio of the number of people engaged in subsistence fishing activity to the number of people employed part or full-time in small-scale fisheries (based on national datasets with sufficient data, rather than datasets with estimates), the number of people engaged in subsistence fishing activity was higher than those workers employed in the fisheries (Fig. S4).

Within countries, detailed sub-national datasets from the surveys in 14 of the 78 countries provide sufficient spatially-linked information to illuminate the extent to which small-scale fisheries' contribution to livelihoods may be highly geographically concentrated. In aggregate, small-scale fisheries in these 14 countries provided 0.6% of the total employment at the national level in 2016, but the harvesting segment of the fisheries provided on average 19% of total employment in 31 coastal and riparian administrative areas (as defined by local government jurisdictions), with an aggregate population of 9.5 million.

II. Detailed methods for global estimates of livelihoods dependent on small-scale fisheries

1. Introduction

Using microdata from 78 household-based surveys (both labor force surveys and household income and expenditure surveys), we developed a conceptual and econometric framework to derive regional and global estimates of livelihoods dependent on small-scale fisheries (SSF) as well as commercial employment in large-scale fisheries (LSF), with breakdown by the pre-harvest, harvest, and post-harvest stages of production. The econometric framework is largely based on weighted multivariate regression analysis and it uses models fit to SSF livelihoods and dependency in 78 countries --where livelihoods and dependency microdata (readily available from surveys) were processed, harmonized and reported at national level-- to produce regional and global estimates of the population engaged in subsistence, commercially employed along the different stages of production in SSF and LSF, as well total dependents upon SSF. The conceptual and statistical framework used for the global predictions of SSF subsistence, employment and dependency is presented in Figure S5. below.

Figure S5. *Conceptual and statistical framework for the global extrapolation of subsistence, employment and dependency in SSF, and employment in LSF.*

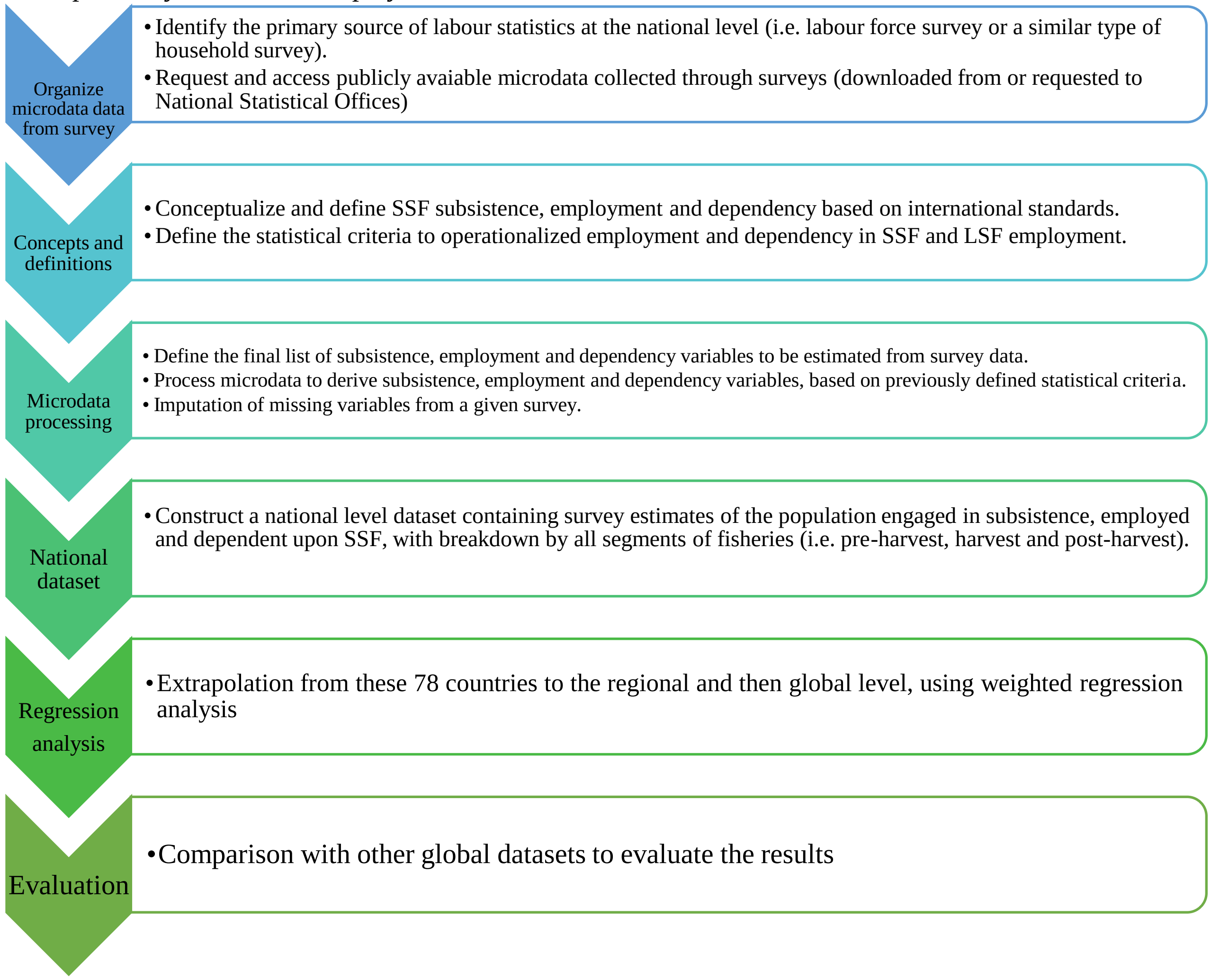

2. Concepts and definitions used

2.1 Subsistence fishing activity

Persons engaged in **subsistence activities** such as farming or fishing (or defined as "working for own consumption"), are not considered as employed according to the ICLS definition (ICLS, 2013). Such persons engage in activities *to produce goods and services for their own final use, such as subsistence food production*. Subsistence fishing activity is defined as that where individuals of any sex and age carry out an activity in order to produce fish for their own final use, with no transaction occurring in the market place (ICLS, 2013). Such individuals are not considered as employed, but do report carrying out a fishing activity at least once over the last 12 months (or for a higher frequency in some surveys) (Table S4).

Table S4. Typology of Small-Scale Fisheries Livelihoods Measured and their Safety Net Function to help prevent poverty

Livelihood Indicator	*Definition*	*Possible Safety Net Function*
Employment	all persons of working age who, during a short reference period, were engaged in any activity to produce goods or services provided for pay or profit, including both part and full-time employment in order to capture seasonal variation	Additional income opportunities for households with low savings, including: (i) serving as a "bank in the water" in times of shocks; (ii) helping to absorb excess unskilled labor; and (iii) forming part of multi-occupational livelihood strategies
Subsistence	working for one's own consumption: workers who produce goods or services which are predominantly consumed by their own household and constitute an important basis for its livelihood	Food production alternatives for households
Livelihoods at least partially dependent upon employment or subsistence	Members of the households of those employed in the fisheries or engaged in subsistence activities, acknowledging that other members may engage in other occupations, and so these are livelihoods at least "partially dependent" upon employment or subsistence	Mix of food production alternatives and additional income opportunities

2.2 Employment

How is the employed population defined? Concepts for employment were defined by the 19th International Conference of Labor Statisticians (ICLS, 2013) as all persons of working age who, during a short reference period, were engaged in any activity to produce goods or provide services for pay or profit. These persons comprise: employed persons "at work", i.e., who worked in a job for at least one hour during the reference period; and employed persons "not at work" due to temporary absence from a job, or to working-time arrangements (such as shifts in work, flextime, and compensatory leave for overtime).[1]

Employed or subsistence workers? This definition distinguishes the concept of employment from the concept of subsistence. The concept of **employment** mainly refers to work "*for pay or profit*" and it implies activities undertaken in *exchange for remuneration in the form of wages or salaries, or in the form of profits* derived from the goods and services produced through market transactions (including remuneration in cash or in kind, whether received or not, and may also comprise additional components of cash or in-kind income).

Defining and operationalizing employment in fisheries. Employment is classified as related to fisheries based on definitions used in the International Classification of Economic Activity (ISIC) standards agreed

[1] ICLS resolution (2013) available at https://www.ilo.org/wcmsp5/groups/public/---dgreports/---stat/documents/normativeinstrument/wcms_230304.pdf.

for use by UN member states (UN, 2015) as a standard by which measures of economic activity can be compared (in the System of National Accounts[2]). In addition to the category of "fishing activities" related to the harvesting stage (divided between marine and inland fisheries by the codes 0311 and 0312 respectively), persons employed in fisheries are classified by different codes for a number of activities related to the pre- and post-harvest stages of production, as indicated in Table S4. While ISIC categories identify persons employed in marine and inland fish harvesting respectively, as well as fish processing, the remaining relevant categories for pre- and post-harvest activities aggregate persons employed in fisheries-related activities together with employment in other sectors (e.g., construction of all types of ships in relation to pre-harvest sub-sector, or wholesale and retail sales of food products in relation to the post-harvest sub-sector). For these categories of pre and post-harvest activities related to fisheries, employment related specifically to fisheries can be identified by cross-checking the ISIC data with categories established under the International Standard Classification of Occupations (ISCO), which identifies jobs by the tasks and duties undertaken.[3] For example, persons employed in industries categorized through ISIC standards as "wholesale of food, beverages and tobacco" can be identified as undertaking work related to the fisheries sector by using the ISCO category further specifying those persons in this ISIC category that are engaged in an occupation concerned with the "wholesale of fishery products." In summary, all employment data recorded and labelled according to the ISIC categories, can be further sub-divided according to ISCO categories, to identify employment related directly to the fisheries sector.

Table S51. ISIC codes to identify employment in fisheries.

Stage of Production	ISIC Code	Industry	Other info for estimating employment in fishery
**Harvest**	**0311**	Marine fishing	
	**0312**	Freshwater fishing	
**Pre-harvest**	**3011**	Building of ships and floating structures	cross-tab with the ISCO classification
	**3319**	Repair of other equipment (it includes repair of fishing nets, including mending)	cross-tab with the ISCO classification
**Post-harvest**	**1020**	Processing and preserving of fish, crustaceans, and molluscs	
	**4630**	Wholesale of food, beverages, and tobacco (it includes wholesale of fishery products)	cross-tab with the ISCO classification
	**4721**	Retail sale of food in specialized stores (it includes fish, other seafood, and products thereof)	cross-tab with the ISCO classification

Disaggregating fisheries employment data between small and large-scale fisheries. While persons employed in activities related to fisheries can be commonly identified in labor statistics across countries based on ISIC and ISCO standards, no distinction is typically made as to whether the employment is linked to small or large-scale fisheries. Currently no universally-accepted definition exists of small-scale fisheries, though common characteristics have been identified, typically based on the scale of harvesting operations (Smith and Basurto, 2019). In the absence of a universal definition, the practice of characterizing operations as small in scale in different sectors has been based on the International Classification of Status in Employment (ICSE, 1993), which classifies employed persons based on their relation to their job, as either paid or self-employed, with the latter sub-divided into "employers", "own-account workers[4]" and

[2] https://unstats.un.org/unsd/nationalaccount/sna.asp
[3] https://www.ilo.org/public/english/bureau/stat/isco/
[4] Own-account workers are those workers who, working on their own account or with one or more partners, hold a 'self-employment job' and have not engaged on a continuous basis any 'employees' to work for them during the reference period. The partners may or may not be members of the same family or household. Own-account workers or unpaid family workers are "often associated with small production units or even subsistence activities" according to ILO (2013b).

“contributing family workers[5].”[6, 7] Using ICSE definitions, persons conducting activities related to fisheries who are identified as “own-account workers” and “contributing family workers” can be assumed to be participating in small-scale fisheries (ILO, 2013, page 57), while those self-employed workers classified as “employers” typically manage a larger-scale enterprise[8] and constantly hire employees (ILO, 2013). In this assessment, persons participating in activities related to fisheries who were characterized in surveys as “own-account workers” and “contributing family workers” were considered to be employed in small-scale fisheries.

The remainder of persons employed in activities related to fisheries were divided between small and large-scale operations based on the total number of workers in the enterprise. Essentially, of those persons considered as employed in activities related to fisheries, workers identified as “own-account workers” and “contributing family workers” are assumed to be participating in small-scale fisheries, and the remainder were identified as participating in either small or large-scale fisheries based on the number of other workers employed in the enterprise. For this study, we assumed that persons considered as employed in activities related to fisheries, who are not classified as “own-account workers” or “contributing family workers”, are participating in small-scale fisheries if the total number of workers in their respective enterprise was below a context-dependent threshold: where **the total number of workers engaged in the production units was lower than two thirds of the 90th percentile number of workers engaged in all fisheries-related enterprises within a given country.** For example, if 90% of fisheries-related enterprises in Ghana have 15 employees or fewer, then all fisheries-related enterprises in the country of fewer than 10 persons were considered as small-scale. The same operational criteria --based on the context-dependent threshold and the status in employment-- was also applied to those who engage in pre and post-harvest activities connected to fisheries as a proxy for operations linked to small-scale harvesting, acknowledging that in some cases large enterprises may process fish caught by small-scale harvesters, and vice versa.

In sum, employment has been generally defined by the ICLS and classified according to common standards that allow for cross-country comparison, according to the type of activity undertaken as defined by ISIC and ISCO standards, as well as the relation of the employee to the job (either paid or self-employed, with the latter sub-divided into “employers”, “own-account workers” and “contributing family workers”). Persons employed in activities related to fisheries, that are classified as “own-account workers” and “contributing family workers” are assumed to participate in small-scale fisheries, and of the remainder, all those persons employed in enterprises whose total number of workers is less than two-thirds of the 90th percentile number of workers engaged in all fisheries-related enterprises within a given country are assumed to participate in small-scale fisheries.

---

[5] Contributing family workers are those workers who hold a 'self-employment' job (cf. paragraph 7) in a market-oriented establishment operated by a related person living in the same household, who cannot be regarded as partners, because their degree of commitment to the operation of the establishment, in terms of working time or other factors to be determined by national circumstances, is not at a level comparable to that of the head of the establishment (ILO, 2013b).

[6] https://www.ilo.org/wcmsp5/groups/public/---dgreports/---stat/documents/normativeinstrument/wcms_087562.pdf

[7] Oya, 2015

[8] The ILO states that: “The term “enterprise” covers a broad variety of business ventures of different sizes (ranging from own-account informal economy operators to multinationals controlling entire global supply chains); ownership structures (family owned, limited company, shareholding company, state-owned or parastatal, as well as cooperatives, mutual benefit societies, and similar social economy ventures); business orientation (commercial enterprises, social enterprises, public enterprises, community enterprises) or economic activity (agricultural, manufacturing, services, or a combination of those).” https://www.ilo.org/global/topics/dw4sd/themes/enterprise-developement/lang--en/index.htm#:~:text=Enterprises%20are%20central%20to%20the,interact%20on%20a%20daily%20basis.

3. Sources of data, processing of data, and methods for global extrapolations

3.1 Sources of data

Ad hoc surveys can easily miss the frequently informal, seasonal nature of the contingent participation of often rural inhabitants in these livelihood-supporting activities. Over the last decade governments worldwide have improved the methodology and coverage of national labor force and household surveys, that better account for seasonal differences in participation in livelihood-supporting activities. Three different types of national household survey instruments have been commonly used to collect data on employment and were used for analysis: population censuses (PCs), labor force surveys (LFS) and household income and expenditure surveys (HIES). PCs are the most comprehensive household surveys conducted at the national level but are less frequently conducted (every 5 to 10 years), and hence less commonly available. Both LFSs and HIESs are large-scale standardized household-based surveys conducted by National Statistical Offices to collect data on a wide array of topics, and although there are differences in scope, both provide the main source of official statistics on a country's labour market indicators.

Labor Force Surveys (LFS). The scope of an LFS is to generate nationally (and in some cases sub-nationally) representative statistics on the working-age population (e.g. employment by sector), based on a survey directed to a sample of households (and all members who are citizens) and conducted through a series of interviews that use a questionnaire drawing upon definitions agreed by the ICLS (see, for example, ILO, 2018).

Household Income and Expenditure Surveys (HIES). The scope of an HIES is broader than the LFS, conducted as household surveys (including all members) aiming to capture a larger set of socio-economic variables ranging from health, education, food and non-food consumption, and sources of income, among others, and includes a module focused on employment. The HIES employment modules do not include as much information as LFS as they are focused more on consumptive expenditures and income (see, for example, Oya, 2015), but they offer a second-best source of data for estimating employment in small-scale fisheries where LFS are not available.

Surveys used in the analysis. To estimate employment and dependence on SSF, the final list of surveys with at least basic information on the sector of employment considered for the analysis includes 34 LFS, 43 household income and expenditure surveys and 1 population census; overall they cover a total of 78 countries. These surveys represented almost 79% of the total World population in 2016 (more than 5.8 billion individuals covered by the surveys) and covered an estimated 78% of world employed population (2016). The available surveys spread over the period 2008-2018, although most survey data have been collected starting in 2014 (55 out of 78 surveys).

Figure S6 below shows the type of survey used for each of the countries analyzed; and Figure S7 shows the share of the world employment population absorbed by the countries used in this study. Unsurprisingly, China and India absorb more than one-third (38.5 %) of the world employment population, followed by the United States, Indonesia, and Brazil. For all these countries, either a LFS or a HIESs was found. Figure S6 shows the geographic distribution of the LFS and HIES used in the analysis.

Figure S6. Countries covered by types of National Survey Instruments used in the analysis (top panel); share of global employment, by country (bottom panel)

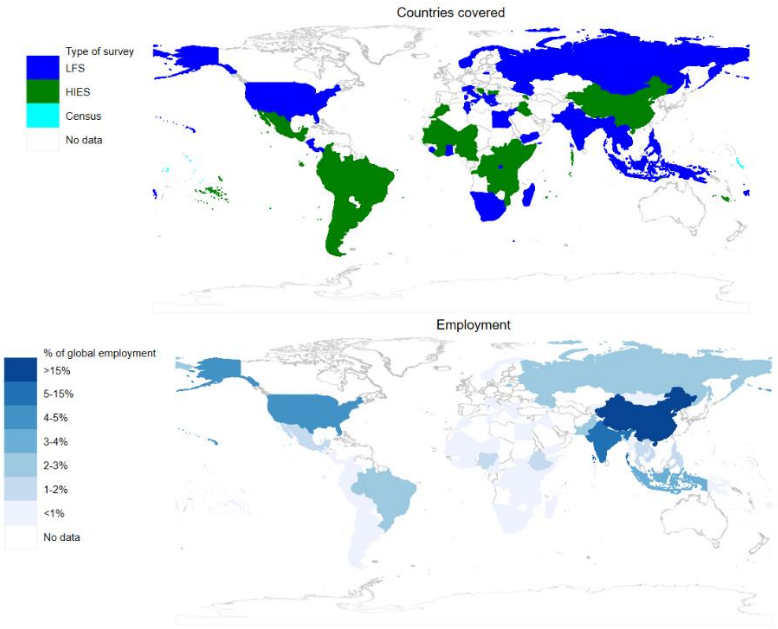

Figure S7. Number of national datasets available for analysis during the study period (2008-2018), by type of household survey instrument and detailed geographic region

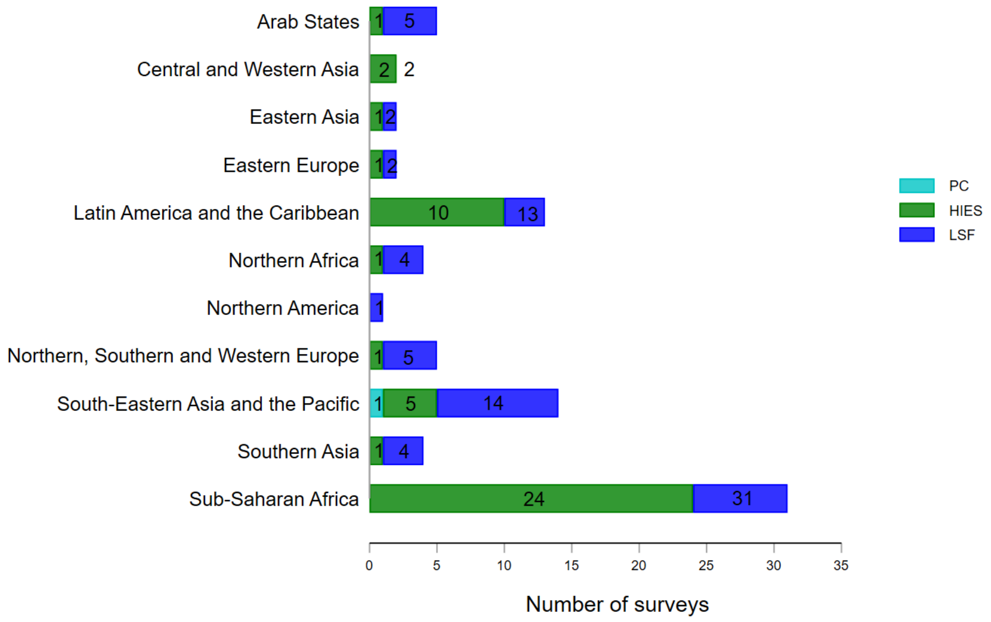

3.2 Data processing and construction of national dataset

Employment, subsistence and dependency-related variables were extracted from these 78 national datasets into one global database (Box S1) with 1336 variables for analysis,

Box S1. Construction of a global database on small-scale fisheries employment, subsistence and dependency

National microdata obtained from 78 national household-based surveys were harmonized by processing the national survey data into a common set of templates. The harmonization process involved the standardization of the key variables used to produce regional and global estimates, including statistical treatment of unspecified counts and a unified assignment of blanks and zeros to missing values. For example, data on employment in marine SSF in countries with no coastlines are converted to zeros, whereas missing counts on employment in inland SSF are treated as missing. The national data sets obtained from surveys have all been processed into a common set of seven templates, which are briefly described below:

- Template 1: Employment population in harvest in SSF and LSF by inland and marine
- Template 2: Employment population in pre-harvest SSF and LSF
- Template 3: Employment population in post-harvest SSF and LSF, by post-harvest processing and post-harvest trading
- Template 4: Population partly dependent upon SSF and LSF
- Template 5: Population fully dependent upon SSF and LSF
- Template 6: Population working in SSF mainly for own-consumption
- Template 7: Population partly dependent upon SSF for own-consumption

Each template is a table of cross-classification of employment and dependency data, with a common column structure defining the small/large scale dimension of the operation unit, as represented in Table below.

Broad Region	Country	Iso_3	Year	Source	Main indicator	Small-scale			Large-scale		
						Marine Harvesting	Inland Harvesting	**Total harvesting**	Marine Harvesting	Inland Harvesting	**Total harvesting**
Sub-Saharan Africa	Cameroon	CMR	2015	Labour Force module attached to HIES	Employment	78,259	39,534	117,792	2,094	0	2,094

This process highlighted some gaps in information across the different survey instruments and national datasets. Information on subsistence fishing activity (i.e., persons who work mainly for their own consumption or for the final consumption of the household) was only available in 11 LFS datasets and 23 HIES, while 43 HIES and LFSs datasets did not include information on pre and/or post-harvest activity in fisheries. In total, the 78 national data sets provided information for the 971 variables at a coverage rate of 72.6 % (see Table S6).

Table S6. Coverage of variables in national datasets.

	Variable	Variable name	Total number of cells	Total number of cells with available information
SSF	Employed population in harvest marine SSF*	MSSF	61	55
	Employed population in harvest inland SSF	ISSF	75	64
	Employment in harvest SSF	HSSF	76	76
	Employed population in pre-harvest SSF	PRESSF	76	31
	Employed population in post-harvest SSF (processing)	POSTSSF	76	45
	Employed population in post-harvest SSF (trading)	POSTSSFT	76	42
	Population partly dependent upon SSF	PDSSF	76	70
	Population fully dependent upon SSF	FDSSF	76	70
	Subsistence work in SSF	WOCSSF	76	34
	Population dependent upon subsistence work in SSF	DWOCSS	76	34
LSF	Employed population in harvest marine LSF*	MLSF	61	52
	Employed population in harvest inland LSF	ILSF	75	64

Employment in LSF	HLSF	76	76
Employed population in pre-harvest LSF	PRELSF	76	31
Employed population in post-harvest LSF (processing)	POSTLSF	76	45
Employed population in post-harvest LSF (trading)	POSTLSFT	76	42
Population partly dependent upon LFS	PDLSF	76	70
Population fully dependent upon LSF	FDLSF	76	70
**Total**	**TOT**	1336	971

Where data for variables were missing in the 78 national datasets, they were imputed, by calculating and applying ratios from the mean of available data from other countries within a geographic archetype, according to the regional grouping provided by the ILO[9]. The most common gaps in various datasets for the 78 countries, and the ratios applied to fill them, were as follows:

- Where distinction between employment in marine and inland small and large-scale fish harvesting activity is not available (*apply ratio of employment in inland small-scale fish harvesting to total employment in small-scale fish harvesting*);

- Where data on employment in pre and/or post-harvest subsectors of fishing activity is not available (*apply ratio of employment in pre and/or post-harvest subsector of fishing activity to total employment in fish harvesting subsector*);

- Where data on dependency on small-scale fisheries is not available (*apply ratio of population fully or partially dependent upon small-scale fisheries to total employment in small-scale fisheries*);

- Where data on subsistence fishing activity ("work for own consumption") is not available (*apply ratio of population engaging in fishing for subsistence to total employment in small-scale fisheries*), or on the population dependent on subsistence fishing activity (*apply ratio of population dependent upon subsistence fishing to total population engaged in fishing for subsistence*);

- Where variables were missing in national datasets more broadly (*apply ratio of missing variable to total employment in fisheries).*

[9] https://ilostat.ilo.org/resources/concepts-and-definitions/classification-country-groupings/

Box S2. Ratios used for imputation of missing variables for the 78 countries with data collected.

Example: distinguishing employment in small and large-scale fish harvesting activity between marine and inland, when not available:

$$\widehat{Emp\_inland}_{i,c,h} = empl\_harvest_{i,c,h} * r_{i,h} \qquad [1]$$

Where $\widehat{Emp\_inland}_{i,c,h}$ denotes the missing count of employment in inland fishery in a given small-scale or large scale operation unit i, for country c in subregion h. $empl\_harvest_{i,c,h}$ represents the total number of persons employed in fish harvesting in a given small-scale or large scale operation unit i, for country c in subregion h. The ratio $r_{i,h}$ is calculated as:

$$r_{i,h} = \frac{\sum_{k \in s_{c,h}} Emp\_inland_{i,c,h}}{\sum_{k \in s_{c,h}} Emp\_harvest_{i,c,h}} \qquad [2]$$

where $s_{c,h}$ is the set of all national data sets in subregion h with non-missing data on inland harvest fishery for small-scale and large-scale operation unit h. It is noteworthy that the ratio $r_{i,h}$ is equal to 1 for landlocked countries (i.e. countries with no coastlines). By contrast, for non-landlocked countries the ratio $r_{i,h}$ is >=0 & <=1

The calculation procedure is exemplified using the numerical example based on the Nigeria GHS-Panel survey conducted in 2014. According to estimates from the Nigeria survey, the size of the employed population in harvest SSF is reported as 1,015,515 in template 1 but the distribution between inland and marine SSF is missing. According to the above imputation --formula [1]-- employment in inland SSF is calculated as:

$$\widehat{Emp\_inland}_{i,c,h} = 1{,}015{,}515 * 0.678 = 688{,}070$$

Where $i = \text{SSF}, c = \text{Nigeria}$ and $h = \text{Sub} - \text{Saharan Africa}$. $r_{i,h} = 0.678$ is the proportion of employment in inland SSF in total employment in harvest SSF, calculated on the basis of all countries in Sub-Saharan Africa with non-missing data on employment in inland SSF. Since Nigeria has coastlines, employment in marine SSF will be non-zero and can be calculated as the difference between total employment in harvest SSF and total employment in inland SSF.

Geographic archetypes were defined to impute missing variables (as described above) at the lowest possible regional grouping, on the basis of the following grouping used by the ILO (geographic archetypes): Arab States, Central and Western Asia, Eastern Asia, Eastern Europe, Latin America and the Caribbean, Northern Africa, Northern America, Northern, Southern and Western Europe, South-Eastern Asia and the Pacific, Southern Asia and Sub-Saharan Africa.

The results in the 78 national datasets were obtained from household surveys conducted between 2008 and 2018, for comparison they were adjusted to the study year 2016, based on annual data from the International Labor Organization (ILO) on the total population employed in either agriculture, forestry or fisheries (i.e. by taking the ratio of employment for the year given from the household surveys to the ILO data on the total population employed in agriculture, forestry and fishery, and applying it to the ILO data for 2016) (Box S3).

Box S3. *Methods for adjusting data in the 78 national datasets to the study year of 2016.*

The results in the 65 national datasets were obtained from household surveys conducted between 2008 and 2018, for comparison they were adjusted to the study year 2016, based on annual data from the International Labor Organization (ILO) on the total population employed in either agriculture, forestry or fisheries (i.e. by taking the ratio of employment for the year given from the household surveys to the ILO data on the total population employed in agriculture, forestry and fishery, and applying it to the ILO data for 2016). This adjustment can be represented by the following:

$$Em\_harvest\widehat{\_fish}\_2016_{i,c,h} = ILO\_emp\_agr\_for\_fish\_2016_{i,c,h} * r_{i,c,h} \quad [1]$$

$$Emp\_pre\_harv\widehat{est\_fish}\_2016_{i,c,h} = ILO\_industry\_2016_{i,c,h} * r_{i,c,h} \quad [2]$$

$$Emp\_post\widehat{\_fish}\_2016_{i,c,h} = ILO\_industry\_services\_2016_{i,c,h} * r_{i,c,h} \quad [3]$$

$$Pop\_dependen\widehat{t\_fis}hery\_2016_{i,c,h} = UNPOP\_2016_{i,c,h} * r_{i,c,h} \quad [4]$$

Where the variables marked with a hat in equation 16-18 denote the adjustted values for 2016 of the population employed in pre-harvest, harvest, and post-harvest fishery, in a given small or large operation unit i, for country c in subregion h, only for those countries with available national data sets. The base population for calibrating the three above-described variables for 2016 are captured by the variables with prefix ILO_*

Similarly, $Pop\_dependen\widehat{t\_fis}hery\_2016_{\ i,c,h}$ in equation 19 denotes the adjusted values of the population partly and fully dependent upon SSF/LSF (index i), for country c, in subregion h for countries with available national data sets. The base population for obtained the calibrated values for the population partly and fully dependent upon SSF/LSF for 2016 are captured by the variable with prefix UNPOP_*

To adjust national household survey data to 2016, ILO and UN data are multiplied by the ratio $r_{i,c,h}$ which captures the proportion of the variable of interest (for example employment in pre-harvest, harvest, post-harvest) related to the year of the survey in total base population corresponding to the year of the survey.

A numerical example illustrates the procedure for adjusting the household survey data in the 77 national datasets to the year 2016. According to estimates from the survey, in 2014, the total employed population in SSF in Nigeria, as extracted from the Nigeria GHS-Panel survey (2014) accounted for 1,015,515 million people. Based on ILO data, the employed population in agriculture, forestry and fishery in 2014 was estimated at 18,789,800. Based on these numbers the ratio $r_{i,c,h}$ is:

$$r_{i,c,h} = \frac{1{,}015{,}515\ (2014)}{18{,}789{,}800\ (2014)} = 0.054$$

According to data extracted from the ILO international repository, in 2016, the total population employed in agriculture, forestry and fishery in Nigeria was estimated at 19,581,800. Therefore, the calibrated values for employment in harvest SSF in Nigeria in 2016 is estimated using formula 1, as follows:

$$Em\_harvest\widehat{\_fish}\_2020_{ssf,nga} = 19{,}581{,}800_{ssf,nga} * 0.054 = 1{,}058{,}319$$

3.3 Global extrapolation: weighted regression analysis

After the harmonization process and the construction of the final dataset at national level, the results from the 78 surveys were extrapolated to the regional level (according to the geographic archetypes used by the ILO), and subsequently to the global level using weighted regression analysis. To correct for non-response bias in countries not included in the 78 national datasets (given that these datasets were selected based on the availability of information, and not randomly as a representative sample of nations worldwide), a weighted regression analysis based on independent variables considered as predictors was used, following recommendations by the ILO (ILO, 2017).

Weights of the different predictor variables were calculated as the inverse probability of selection (or inverse propensity score), to account for differences between the 78 countries for which data was collected, and the world's remaining countries to which the results were extrapolated. For example, per capita GDP is lower, total population is higher, and the portion of the employed population participating in agriculture, forestry and fisheries is higher on average in the 78 countries for which data was collected, compared to those for whom it was not (Table S7)

Table S7. Key socio-economic differences between reporting and non-reporting countries

	Reporting		Non-reporting countries	
**Key socio-economic indicators**	Mean	Median	Mean	Median
Share of employment in agriculture, forestry and fishery (% of total employment)	44.0%	42.3%	38.1%	40.8%
Per-capita GDP, 2015 (2017 International $)	5,566	3,849	14,503	4,700
Value added in agriculture, forestry and fishery (% of GDP)	21.3 %	18.6 %	15%	11.7%
Total population (million)	143,000	35,100	18,500	7,244

To account for these differences (i.e. non-response bias), the country's probability of reporting employment in small-scale fisheries was estimated as the function of a set of covariates (Crespi, 2004), based on a cumulative standard logistic distribution, using maximum likelihood estimation, as follows:

$$\Pr(Y = 1 \mid X_{1,} X_{2,} \ldots X_K) = \frac{1}{1 + (\frac{1}{e^{(\beta_0 + \beta_0 X_1 + \beta_1 X_1 + \cdots + \beta_k X_K)}})} \quad [6]$$

On the basis of [6], a given country's probability of reporting small-scale fisheries employment was estimated, using four predictor variables identified based on assessment of their explanatory power for estimating employment and dependency: (i) % of a country's GDP generated from agriculture, forestry and fisheries; (ii) a country's population size; (iii) a country's per capita GDP; and (iv) a variable indicating the country's regional archetype, given as follows:

$$\Pr(R_{c,r} = 1) = Pr(x_{c,r}\beta, + \varepsilon_{c,r}) \quad [7]$$

Where c, r are indices for country and broad region; $R_{c,r}$ is equal to 1 if the country reports employment in small-scale fisheries and 0 otherwise; $x_{c,r}$ is a vector of the independent variables expected to influence the probability to report employment in small-scale fisheries and $\varepsilon_{c,r}$ is the usual error term. The results provide the predicted response probabilities for each country within each geographic archetype, used to calculate the weights for the regression, defined as follows:

$$W_{c,r} = \frac{\Pr(R_{c,r} = 1)}{(\frac{Reporting\ countries}{total\ countries})} \quad [8]$$

Where the final weight is calculated as the ratio between the estimated country's probability over the proportion of reporting countries in total countries in the data set. Using these weights to correct for non-response bias, a weighted regression analysis was conducted to extrapolate from the 78 national datasets to regional and global estimates, essentially generating estimates based on assumed relationships between employment and dependency variables and a set of predictor variables.

The specification of the regression analysis was implemented by using a cross-country data set with the inclusion of a categorical variable to control for potential heterogeneity across (broad) regions. The sample of reporting countries was weighted using $w_{c,r}$.

In general, the following linear equation [9] was implemented and run on a set of dependent variables capturing employment and dependency in fisheries:

$$\widehat{Y_c} = \alpha_r + x_c'\beta + \varepsilon_i \quad [9]$$

Where $\widehat{Y_c}$ denotes the variable to be estimated for employment and dependency, as specified in the first row of Table S4, for country c; $\alpha_r$is a categorical variable which captures the broad sub-region; x_c' is a set of predictor variables of employment in small and large-scale fisheries and dependency upon small and large-scale fisheries that are expected to influence the outcome variables (Table S8); $\varepsilon_i$is the error term, assumed to be normally distributed. The choice of the predictor variables for estimation purposes (Table S9) is guided by two considerations: first, the predictor variables must be strongly correlated with the outcome variables to be estimated, in order to ensure a high explanatory power (measured by means of the R squared) of the corresponding regressions. For example, the correlation between employment in inland and marine small-scale fisheries and their corresponding **main** predictor variables is shown in Table S8 below. Second, the selected predictor variables must be available for the largest number of countries in the global data set, so that regional and global estimates of employment and dependency data are based on a vast majority of countries throughout the world.

Table S8. Observed correlation between employment in inland and marine SSF and their corresponding predictor variables.

	Employment in inland SSF					
Employment in inland SSF	1					
Area of inland water bodies	0.7604*	1				
Employment in agriculture, forestry and fishery	0.8168*	0.6135*	1			
GDP per-capita (PPP)	-0.2723*	0.1362	-0.3296*	1		
GDP growth	0.2707*	0.0765	0.2588*	-0.3090*	1	
Value added in agriculture forestry and fishery	0.1005	-0.018	0.3100*	-0.6016*	0.218	1
	Employment in marine SSF					
Employment in marine SSF	1					
length of coastline (Km.)	0.6505*	1				
Employment in agriculture, forestry and fishery	0.7509*	0.3459*	1			
GDP per-capita (PPP)	-0.0972	0.4790*	-0.3296*	1		
GDP growth	0.066	-0.2013	0.2588*	-0.3090*	1	
Value added in agriculture forestry and fishery	0.0133	-0.1678	0.3100*	-0.6016*	0.218	1

Table S9. List of predictor variables to estimate employment in SSF and dependency upon SSF (including the sub-elements) in non-reporting countries.

		Employment in $\widehat{Y}_c$										Subsistence work $\widehat{Y}_c$	Dependents on: $\widehat{Y}_c$				
		SSF					LSF					SSF	SSF			LSF	
**Explanatory variables** (x_c')	Source	Pre-harvest	Harvest marine	Harvest inland	Processing	Trading	Pre-harvest	Harvest marine	Harvest inland	Processing	Trading		Full	Partial	Partial subsistence	Full	Partial
Employment in agriculture, forestry and fishery	ILOSTAT		X	X				X	X				X	X		X	X
Employment in industry	ILOSTAT	X			X	X	X			X	X						
Employment in services	ILOSTAT	X			X	X	X			X	X						
Total population	UN estimates and projections												X	X	X	X	X
Real GDP per-capita expressed in international dollar (PPP)	World Bank (WDI)	X	X	X	X	X	X	X	X	X	X						
Real GDP growth rate	World Bank (WDI)	X	X	X	X	X	X	X	X	X	X						
Share of value added in agriculture, forestry and fishery (% of GDP)	World Bank (WDI)		X	X				X	X			X					
Share of value added in industry (% of GDP)	World Bank (WDI)	X			X	X	X			X	X						
Share of value added in services (% of GDP)	World Bank (WDI)	X			X	X	X			X	X						
Capture fishery production (metric tons)	FAO-Fishstatj	X			X	X	X			X	X	X			X		
Length of country coastline (in Km.)	GIS data (Natural Earth)		X					X				X			X		
Area (in squared Km) of major inland water bodies and reservoirs	GIS data (Global Lakes and Wetlands Database)			X					X			X			X		

Finally, Box S4 below explains the procedure applied to extrapolate gender-disaggregated statistics for employment in SSF at regional and global level.

Box S4. *Method for the global extrapolation of gender-disaggregated variables.*

The 78 national datasets created from the household-based surveys contain information on the sex of the individual who participates in any SSF/LSF related activity, namely pre-harvest, harvest or post-harvest. In order to capture the share of women who participate in each SSF/LSF activity, the employment variables extracted from the 78 national datasets were gender disaggregated.

The gender distribution in each SSF/LSF activity was extrapolated to the regional level (according to the geographic archetypes used by the ILO), and subsequently to the global level. Regional and global estimates based on partial coverage of all countries is achieved by imputing values for the non-reporting countries (i.e. countries where national datasets are not available) using the information from countries with available national datasets and, finally, aggregating the results to regional and global totals.

The approach used for the regional and global estimates of women's participation in SSF/LSF was to treat the countries with available datasets as a sample of countries selected. The imputation method adopted for regional and global extrapolation was based on three interrelated steps: 1) computation of gender ratios from the available 76 national datasets; 2) extrapolation of the average gender ratio from the sample of national datasets at the lowest possible regional grouping, according to the ILO regional grouping; 3) finally, imputation of missing gender-disaggregated data in countries with not available datasets by applying the average gender ratio to the corresponding employment variable derived from the econometric specification (see section "method used"). Formula [1] below provides a useful example of the imputation of missing gender-disaggregated data for employment in harvesting SSF:

$$Wom\_Emp\_SSF\_harvest_{c,h} = \widehat{Emp\_SSF\_harvest}_{c,h} * r_h \qquad [1]$$

r_h is the average share of women (from 0 to 1) who engage in harvesting SSF in region h, calculated from the available national datasets for region h. The total number of women who engage in harvesting SSF in country c and region h is calculated by applying the coefficient r_h to the estimated total employment in harvesting SSF.

4. Data checks on estimates of fisheries employment

Comparison of results with country case studies conducted for the Illuminating Hidden Harvests (IHH) assessment. The estimates of employment in the harvesting segment of small-scale fisheries value chains generated from national household-based surveys were further compared to the harvesting employment figures of 48 out of 58 IHH country case studies (CCSs) with reported data on employment in small-scale fisheries.

Figure S8 below shows the correlation between employment numbers generated from household surveys and country IHH case study numbers (panel a), as well as country-specific absolute differences between household surveys and IHH country case study employment numbers (panel b), for the harvesting small-scale fisheries sector. More specifically, the vertical axis in panel b (Figure S8) measures the absolute difference between household surveys data for the harvesting segment of small-scale fisheries and the corresponding IHH country case study numbers, while the horizontal axis presents the individual countries

for which employment data for the harvesting small-scale fisheries sub-sectors (both marine and inland) are available from both sources.

The two sources, i.e., household-based surveys and IHH country case studies, show similar results. Estimates of employment in the harvesting segment of the marine small-scale fisheries value chain from household survey data points to 5.7 million employed while, for the same countries and category, country case study data points to 5.8 million people employed. In absolute terms, the largest difference was the case of Vietnam, where employment estimates generated from survey data were larger by a factor of 281 thousand people compared to employment data collected through the country case study (Figure S8, panel b, upper graph). Comparison of employment estimates for inland small-scale fisheries harvesting with the data from 24 IHH country case studies where employment data were available, showed similar results: an estimated 3.7 million people employed compared to 4.6 million in the case studies, with this difference largely imputable to the cases of Nigeria and Indonesia (Figure S8, panel b, lower graph).

Figure S8. Comparison of survey estimates of employment in the harvesting segment of small-scale fisheries, with data from the country case studies

a. correlation

b. difference between household survey estimates and IHH CCSs data

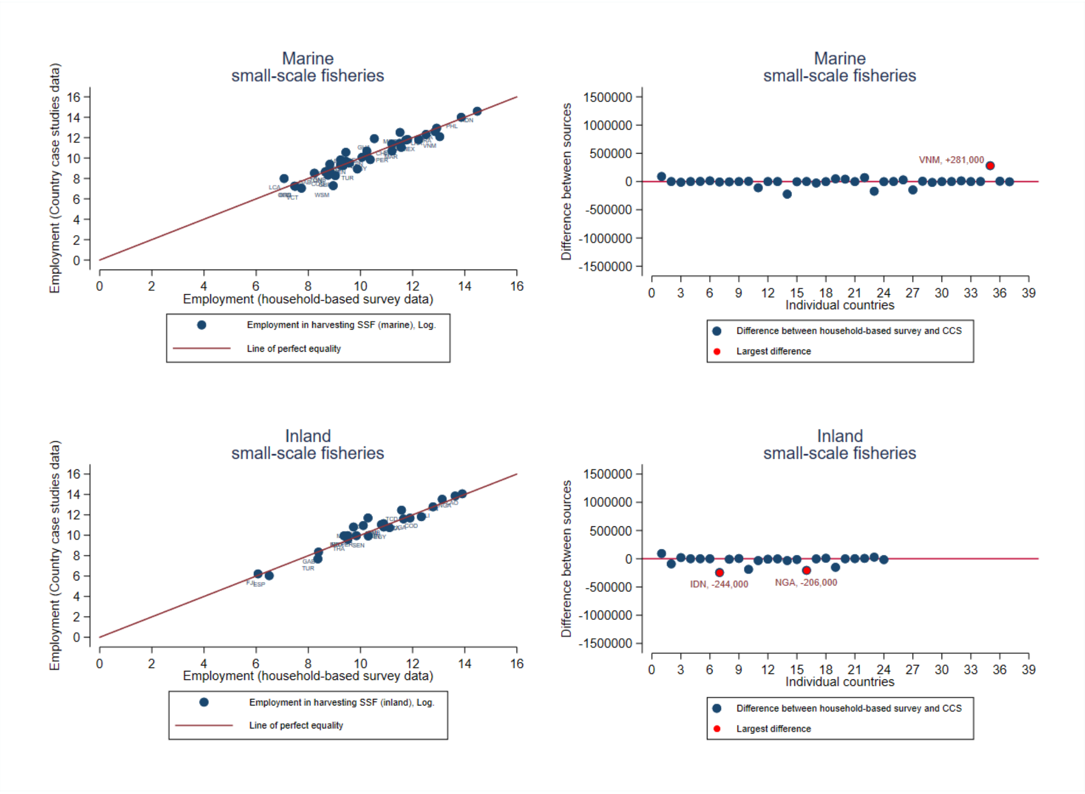

Sources: Household-based surveys and IHH country case studies.

Comparison with FAO aggregated employment data. The estimates of employment in the harvesting stage of fisheries production generated from the national household-based surveys in 78 countries, were compared with FAO FishStatJ employment data.

Figure S9 below shows the correlation between FAO and household survey estimates (panel a), as well as country-specific absolute differences between household survey and FAO employment numbers (panel b). As before, the vertical axis in panel b (Figure S9) measures the absolute difference between household survey employment data for the whole harvesting segment of capture fisheries value chains and the corresponding FAO numbers, while the horizontal axis presents the individual countries for which employment data are available from both sources.

The two sources of data for marine fisheries were highly correlated (correlation=0.90) (Figure S9, panel a, upper graph), with only a handful of countries that show significant differences between the two sources, e.g. China, Brazil and the Philippines (Figure S8, panel b, upper graph). For the case of China, that largest difference is probably since "not all data reported by China [to FAO] is separated between fish farmers and fishers and so some of the fish farmers may be aggregated with fishers."[10]

Similarly, estimates of employment in the harvesting sector of inland fisheries generated in this study were also highly correlated with FAO employment data for that segment (correlation = 0.92), with only a handful of countries showing large differences between the two sources (Figure S9, panel b, lower graph), for example, Cambodia, Myanmar, Nigeria.

Figure S9. Comparison of estimates of employment in the harvesting segment of fisheries, with FAO data

a. correlation b. difference between IHH and FAO data

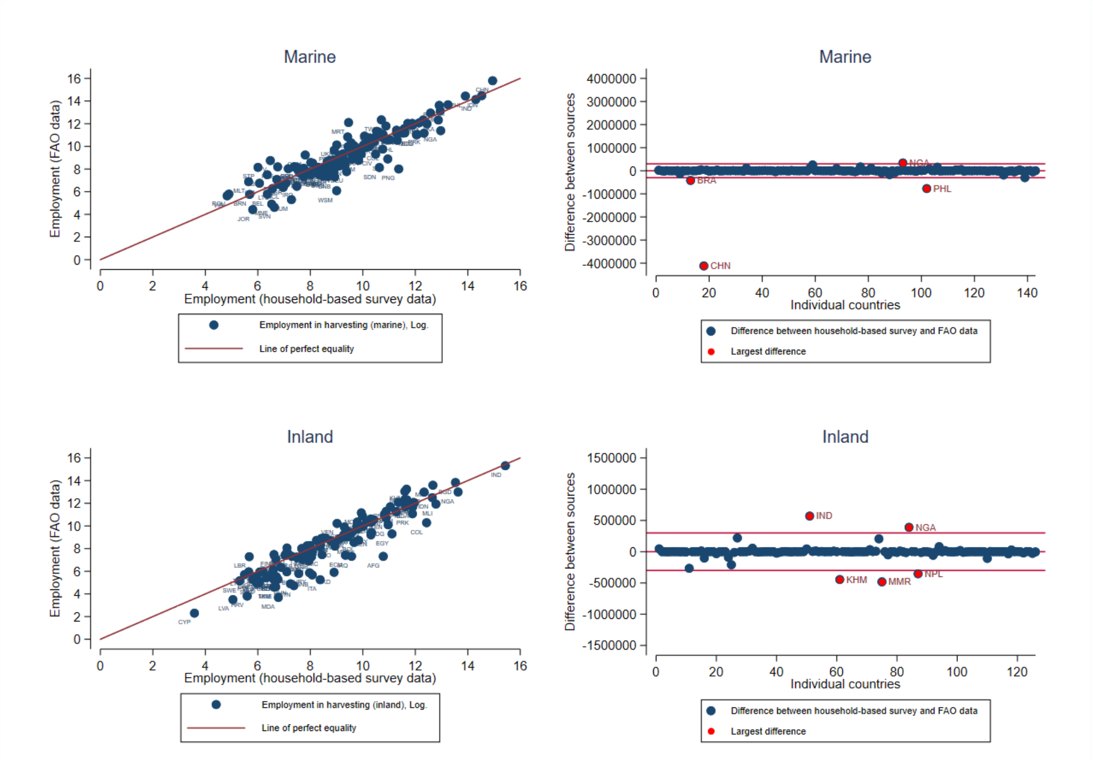

Sources: Household-based surveys and FAO.

---

[10] FAO. 2018. Fishery and Aquaculture Statistics: Annual Yearbook 2018. FAO: Rome. Available at: https://www.fao.org/fishery/static/Yearbook/YB2018_USBcard/booklet/web_CB1213T.pdf

Additionally, estimates of employment in the harvesting stage of fisheries were correlated with FAO data on total capture fisheries production, finding a high and significant (0.86) correlation (Figure S10). The data show a positive and **nonlinear relationship** between the estimates of employment in the harvesting segment of fisheries and total capture fisheries production, suggesting that increases in production are associated with increases in employment up to a point, after which total capture fisheries production continues to increase but less than the increase in employment (i.e. as fish production increases, lower increases in employment are required for marginal increases in production).

Figure S10. Correlation between estimated employment in the harvesting segment of fisheries, and FAO data on total capture fisheries production (log-transformed values).

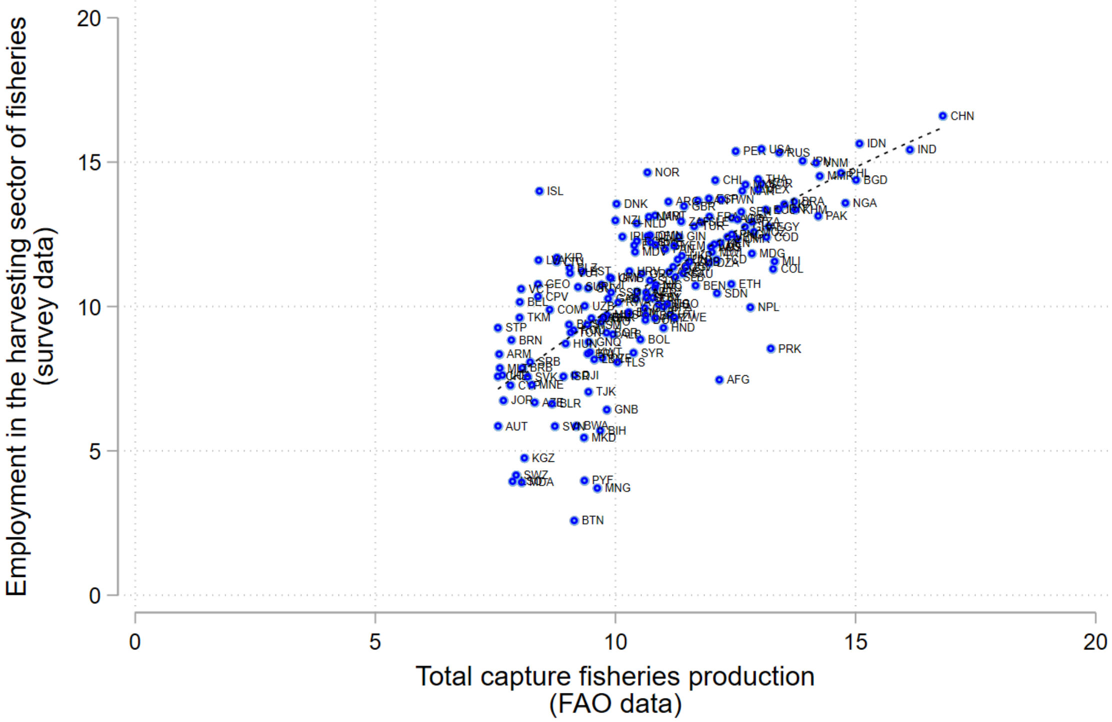

Sources: Household-based surveys and FAO.

III. Detailed methods for estimates of the nutritional contribution of subsistence catch in 14 country case studies

Country case studies with estimates of subsistence catch volume. The IHH assessment included 58 country case studies, where researchers compiled available data on a wide range of indicators of the contributions of small-scale fisheries to society for the years 2013-2017, including the volume of catch landed by species. For the catch, researchers were asked to draw upon existing data or expert judgement in order to estimate the percentage of the volume of each species landed that was used for commercial human consumption domestically, commercial export, subsistence, or non-human consumption (FAO et al. forthcoming). This data on the utilization of the catch was only available for 26 of the countries, and of these only in 14 was subsistence catch reported, in order to estimate the average total volume of the annual subsistence catch by species, or in some cases by groups of species, for both marine and inland fisheries for the period from 2013 through 2017 (multiplying the volume of catch landed for each species or species group, by the percentage reported or estimated to be used for subsistence) (Figure S11).

Figure S11. Average share of catch used for subsistence (average 2013-2017) by country and subsector (marine and inland). **Source:** Illuminating Hidden Harvest country case study data.

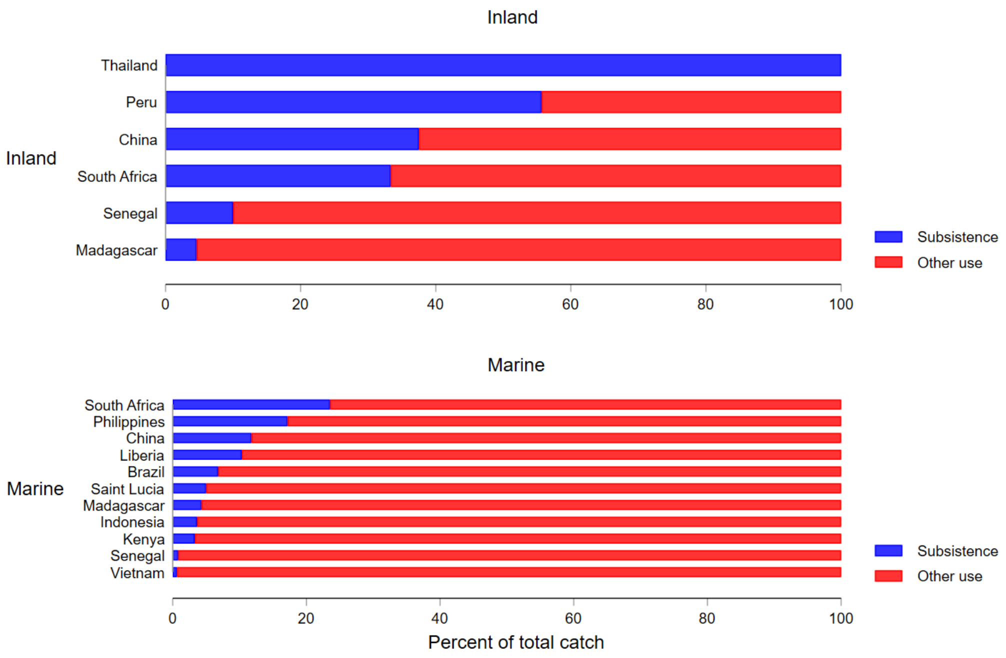

Livelihoods supported by the subsistence catch volume in the 14 countries. For each of the 14 countries, the estimated volume of subsistence catch was assumed to be consumed equally by the number of persons estimated to be engaged in subsistence fishing and their household members, according to the national household surveys used to estimated small-scale fisheries workers. On this basis, the estimated subsistence catch was converted into grams and divided by the total number of persons estimated to be engaged in subsistence fishing plus their household members, in order to generate an estimated per capita apparent consumption of subsistence catch by species, for a total number of persons. This provided a coarse measure of the average per capita apparent fish consumption from the estimated subsistence catch.

Estimating the nutrition contribution of the apparent fish consumption from subsistence catch. Drawing upon nutrient data compiled from peer-reviewed publications and existing databases for over 500 marine and inland fish species, the Illuminating Hidden Harvests assessment developed a model for predicting the composition of the species of six nutrients commonly deficient and driving poor health outcomes: iron, zinc, selenium, calcium, vitamin A and omega-3 fatty acids (FAO et al. forthcoming). Predictions from models are publicly available online through Fish Base (MacNeil et al., 2021). The nutrient values (per 100 grams of fish) at fish species level for the six nutrients mentioned above were matched with data capturing the estimated per capita daily intake of fish from subsistence catch at the same resolution (i.e. by species).

A significant volume of the catch in each of the 14 countries was not identified by species ("not elsewhere included" or NEI), where the detailed or major functional group of the species was identified in the catch data, but not the specific name of the species. For this subsistence catch labeled NEI, the nutrient composition imputed based on the mean nutrient values of species caught by marine or inland small-scale fisheries within the same country. The basic form of imputation for outliers was based on deductive methods and it involved using logical relationships to derive a value for the missing nutrient value. The imputation method was largely based on "donor method" and implemented by assigning the value from a set of records with an observed item (the donor), to a record with a missing nutrient value on that item (the recipient). Thus, missing nutrient data for a recipient fish species was gap filled with the mean value from a donor that had similar characteristics. To select the best donor, many "imputation classes" were constructed, with classes defined. in a hierarchical order, based on country, small-scale fishery sub-sector (i.e. marine and inland) and detailed and major functional groups (Table S11). Within each class, the mean value was then calculated from non-missing nutrient values. Table S11 below shows the output of the imputation procedure. Almost three-quarter (71.2%) of total missing nutrient values were gap filled using the mean nutrient values of species belonging to the same country, sector (marine or inland) and detailed (or major) functional group of species. An additional 25.8% of missing nutrient values was gap filled using the mean nutrient value of species belonging to the same sector and detailed functional group. Finally, only 3% of missing nutrient values was gap filled using the mean value of species belonging to the same country and sector.

Table S11. Imputation of missing nutrient values by hierarchical imputation classes

Level of imputation	Class	Total number of nutrient values gap filled	% of total missing nutrient value
1st	Country, sector, and detailed functional group	74	56.06
2nd	Country, sector, and major functional group	20	15.15
3rd	Sector and detailed functional group	34	25.76
4th	Sector and major functional group	0	0
5th	Detailed functional group	0	0
6th	Major functional group	0	0
7th	Country and sector	4	3.03
8th	Sector	0	0
9th	Country	0	0
	**Total gap filled nutrient values**	**132**	**100**

Following the imputation and matching procedure explained above, the daily per-capita apparent nutrient contribution of fish from subsistence catch, based on 100 grams of fished consumed, was calculated for six key nutrients, as follows:

$$\begin{cases} NC\_calcium_s = Fish\_intake_s * Nutrient\_calcium_s/100 \\ NC\_selenium_s = Fish\_intake_s * Nutrient\_selenium_s/100 \\ NC\_omega\_3_s = Fish\_intake_s * Nutrient\_omega3_s/100 \\ NC\_zinc_s = Fish\_intake_s * Nutrient\_zinc_s/100 \\ NC\_Vitamin\_a_s = Fish\_intake_s * Nutrient\_Vitamin\_a_s\ /100 \\ NC\_iron_s = Fish\_intake_s * Nutrient\_iron_s/100 \end{cases} \qquad \textbf{[1]}$$

Where index s captures the fish species. For each of the six nutrients --iron, zinc, selenium, calcium, vitamin A, and omega-3 fatty acids-- the nutrient contribution (“$NC\_ *_s$”) was calculated by multiplying the average per capita daily intake from the *s-th* fish species by the corresponding nutrient value ($Nutrient\_ *_s$) for the same fish species “s”. This calculation allowed generating the absolute per-capita nutrient intake (expressed in milligram per day) from each species that form the volume of marine and inland subsistence catch, for six key nutrients.

Comparing the average apparent per capita consumption of six nutrients from the estimated subsistence catch in 14 countries, to the recommended daily intake. To better visualise the nutrient contribution from subsistence catch, the estimated values for the average apparent per capita consumption of six nutrients from the subsistence catch in the 14 countries were compared to the minimum recommended nutrition intakes (RNI) for the same six nutrients. The RNIs for Calcium, Selenium, Zinc, Iron and Vitamin A were based on the WHO/FAO “Vitamin and mineral requirements in human nutrition[11]”; for Omega-3 fatty acid, the RNI was based on the “Nutri-Facts[12]” website, which offered scientifically substantiated facts on vitamins, carotenoids and micronutrients, including Omega-3 fatty acid. Since the two sources provided the RNI for different age and sex profiles, the average RNI was calculated by averaging the RNI of different profiles, as highlighted in green in Table S12 below.

Of note, nutrient intakes from the subsistence catch were capped at 100%, such that if 100g of a given fish, or fish catch, provided ≥100% of the recommended nutrient intake for all 6 nutrients, that species or catch would attain a score of 600% (100% x 6 nutrients).

[11] https://apps.who.int/iris/bitstream/handle/10665/42716/9241546123.pdf?sequence=1&isAllowed=y

[12] https://www.nutri-facts.org/en_US/nutrients/items/essential-fatty-acids/essential-fatty-acids/intake-recommendations.html

Table S12. Recommended nutrition intakes (RNI) for different age and sex profiles. Source: WHO-FAO "Vitamin and mineral requirements in human nutrition" and "Nutri-Facts".

Category	Sex	Age	**Calcium** (mg/day)	**Selenium** (mcg/day)	**Zinc** Moderate bioavailability (mg/day)	**Iron** Bioavailability of 10% (mg/day)	**Vitamin A** Recommended safe intake (mcg RE/day)	**Omega3-Fatty acid** g/day
Infants	Both	0-6 months	350	6	2.8		375	0.5
Infants	Both	7–12 months	400	10	4.1		400	0.5
Infants	Both	0-1 year				9.3		
Children	Both	1–3 years	500	17	4.1	5.8	400	0.7
Children	Both	4–6 years	600	22	4.8	6.3	450	
Children	Both	4-8 years						0.9
Children	Both	7–9 years	700	21	5.6	8.9	500	
Children	Females	9-13 years						1
Children	Males	9-13 years						1.2
Adolescents	Females	11-14 years				32.7		
Adolescents	Females	11-14 years, Pre-menarche				14		
Adolescents	Males	11-14 years				14.6		
Adolescents	Females	14-18 years						1.1
Adolescents	Males	14-18 years						1.6
Adolescents	Females	10-18 years	1300	26	7.2		600	
Adolescents	Males	10-18 years	1300	32	8.6		600	
Adolescents	Females	15-17 years				31		
Adolescents	Males	15-17 years				18.8		
Adolescents	Females	18+ years				29.4		
Adolescents	Males	18+ years				13.7		
Adults	Females	19+ years	1000	26	4.9		500	1.1
Adults	Females	51-65 years	1300	26	4.9		500	
Adults	Males	19 + years						1.6
Adults	Males	19-65 years	1000	34	7		600	
Elderly	Females	65+ years	1300	25	4.9		600	
Elderly	Males	65+ years	1300	33	7		600	
Postmenopausal	Females					11.3		
Pregnant	Females	First trimester			5.5		800	1.4
Pregnant	Females	Second trimester		28	7		800	1.4
Pregnant	Females	Third trimester	1200	30	10		800	1.4
Lactating	Females					15	850	1.3
Lactating	Females	0-3 months	1000	35	9.5			
Lactating	Females	4-6 months	1000	35	8.8			
Lactating	Females	7-12 months	1000	42	7.2			
**RNI**	**Average across age and sex profiles**		**953.1**	**26.4**	**6.3**	**16.2**	**585.9**	**1.1**

Panel B of Figure S12 below shows the average nutrient contribution for each functional group of species of the subsistence catch, calculated by averaging the percentage contribution to the RNI of the six nutrients in each functional group. Therefore, each bar shows the average contribution of the six nutrients to the RNI, across all species that form each functional group.

Figure S12. Panel A: Average contribution of marine and inland subsistence catches to the daily RNI for six nutrients. Each bar is the mean RNI contribution of a given nutrient across all species that comprise each group of fishes. Panel B: mean of six nutrients, calculated by averaging the percent contribution to the RNI of the six nutrients presented in Panel A. **Source:** Subsistence catch based on IHH country case studies; estimates of total livelihoods dependent based on national household survey data; nutrients values for six nutrients based on predictions from data available online through Fish Base.

Panel A **Panel B**

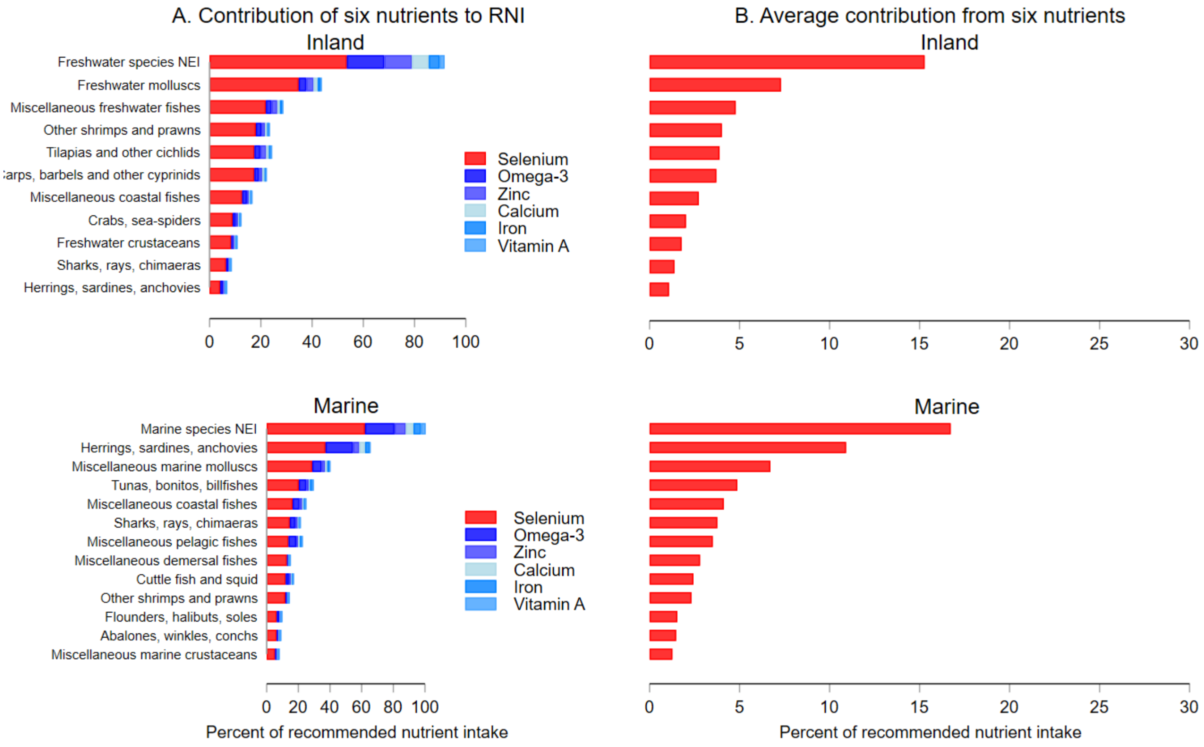

The average apparent contribution of subsistence catches to the daily RNI for the livelihoods dependent upon subsistence activities in inland and marine small-scale fisheries in the 14 countries were calculated by averaging the mean contribution of the six nutrients to the RNI, across all species that form each functional group. The final calculated metric captures, therefore, the average contribution to the daily recommended RNI provided across all species that form a given functional group, not the absolute mean nutrition contribution provided by all species that form subsistence catch. While the analysis of the nutrient contribution of subsistence catch to the daily RNI carried out at the level of group of species (i.e. functional groups) offers a useful indication to understand which group of species contribute the most to achieve the daily RNI for the population dependent upon subsistence activities in small-scale fisheries, it does not provide any indication about "how much" the aggregate volume of subsistence catch contributes, on average, to the apparent per-capita daily intake of the six key nutrients (relatively to the RNI).

To better understand the contribution of the aggregate volume of subsistence catch to the RNI, the per-capita nutrition contribution to the daily RNI provided by the apparent intake of all fish species that form

the aggregate volume of subsistence catch in 14 countries was calculated. The daily per-capita apparent contribution to the RNI provided by all fish species that form the aggregate volume of subsistence was calculated separately for the marine and inland subsistence sector. This was done by summing the nutrition values (for six key nutrients) across all fish species for which we knew the corresponding volume of marine and inland subsistence catch, and then dividing the total by the daily RNI.

In virtually all countries, the contribution of subsistence catches to the daily RNI was mainly driven by fish species with a higher concentration of selenium, omega-3 and zinc, and to a lesser extent of iron, contributing, on average, by 81.7%, 35.1%, 10.3% and 8.8 to the daily RNI (Table S12). Subsistence catches of inland species contain an even higher concentration of selenium and zinc compared to the marine sector, thus supporting the predominant role of inland subsistence activities to a healthy diet for the population dependents upon inland subsistence activities. Omega-3 was found in higher concentration among marine species.

Table S13. Average contribution of marine and inland subsistence catches to the daily RNI for six nutrients. Each column represents the mean RNI contribution of inland and marine subsistence catch for the corresponding nutrient. Note: Recommended nutrient intakes are capped at 100%, such that if 100g of a given fish, or fish catch, provided ≥100% of the recommended nutrient intake for any of the 6 nutrients, that catch would attain a score of 100%. **Source**: Subsistence catch based on IHH CCSs; IHH estimates of total dependents based on survey data; nutrients values for six nutrients based on predictions from data available online through Fish Base.

	Selenium	Omega-3	Zinc	Calcium	Iron	Vitamin A	Average	Volume of subsistence catches (tons/year)	Livelihoods dependent upon subsistence fishing
**% Contribution to daily RNI**									
**Marine and inland subsistence fishing**									
Brazil	26.0	5.3	3.2	1.9	3.1	0.4	**6.7**	2,064	510,006
China	100	22.7	14.7	8.7	6.2	3.4	**26.0**	2,621,888	93,000,000
Ghana	100	28.4	21.1	12.8	8.2	3.7	**29.0**	6,292	172,748
Indonesia	100	58.3	21.4	13.5	12.1	6.9	**35.4**	250,556	4,956,744
Kenya	42.9	6.1	3.2	0.7	0.7	3.5	**9.5**	748	69,898
Liberia	100	100	27.5	22.1	15.6	2.3	**44.6**	1,440	29,920
Madagascar	88.1	10.8	5.8	3.9	2.8	0.7	**18.7**	8,012	743,894
Peru	100	27.0	33.1	11.4	12.4	5.6	**31.6**	2,182	51,204
Philippines	100	46.1	24.6	20.1	12.1	4.2	**34.5**	202,423	4,900,726
Saint Lucia	100	66.4	24.2	15.9	23.8	10.5	**40.1**	276	3,021
Senegal	100	58.5	20.4	14.2	13.6	3.7	**35.1**	12,258	318,156
South Africa	62.6	31.4	8.5	5.0	5.4	2.7	**19.3**	3,594	124,141
Thailand	100	24.8	18.1	12.7	6.0	4.0	**27.6**	203,790	6,079,128
Vietnam	24.1	6.1	1.8	1.2	1.5	0.6	**5.9**	8,257	1,562,177
**Average/Total**	**81.7**	**35.1**	**16.3**	**10.3**	**8.8**	**3.7**	26.0	3,323,780	112,521,763
Inland subsistence fishing									
China	100	20.6	20.3	11.4	7.3	4.1	**27.3**	1,802,486	54,900,000
Ghana	100	28.4	21.1	12.8	8.2	3.7	**29.0**	6,292	172,748
Madagascar	76.2	8.7	6.8	4.1	2.6	1.1	**16.6**	2,130	201,567
Peru	100	27.0	33.1	11.4	12.4	5.6	**31.6**	2,182	51,204
Senegal	100	28.7	22.8	14.1	9.4	3.0	**29.7**	2,683	73,529
South Africa	25.2	2.5	1.4	1.2	0.4	0.3	**5.2**	189	73,886
Thailand	100	24.8	18.1	12.7	6.0	4.0	**27.6**	203,790	6,079,128
**Average/Total**	**85.9**	**20.1**	**17.7**	**9.7**	**6.6**	**3.1**	**23.8**	**2,019,753**	**61,552,061**
Marine subsistence fishing									
Brazil	26.0	5.3	3.2	1.9	3.1	0.4	**6.7**	2,064	510,006
China	100	24.7	9.2	5.9	5.2	2.8	**24.6**	819,402	38,100,000
Indonesia	100	58.3	21.4	13.5	12.1	6.9	**35.4**	250,556	4,956,744
Kenya	42.9	6.1	3.2	0.7	0.7	3.5	**9.5**	748	69,898
Liberia	100	100	27.5	22.1	15.6	2.3	**44.6**	1,440	29,920
Madagascar	100	12.9	4.9	3.7	2.9	0.4	**20.8**	5,883	542,327
Philippines	100	46.1	24.6	20.1	12.1	4.2	**34.5**	202,423	4,900,726
Saint Lucia	100	66.4	24.2	15.9	23.8	10.5	**40.1**	276	3,021
Senegal	100	88.4	18.0	14.3	17.8	4.5	**40.5**	9,575	244,627
South Africa	100	60.4	15.7	8.8	10.4	5.1	**33.4**	3,404	50,256
Vietnam	24.1	6.1	1.8	1.2	1.5	0.6	**5.9**	8,257	1,562,177
**Average/Total**	**81.2**	**43.1**	**14.0**	**9.8**	**9.6**	**3.7**	**26.9**	**1,304,029**	**50,969,701**

Estimates of the nutrient contribution of meat and poultry to the RNI. For the same fourteen countries for which we estimated the nutrient contribution of subsistence catch to the RNI, we used data from FAOSTAT [13]and USDA food composition database[14] to estimate the nutrient contribution to the RNI of beef and poultry. For each of the fourteen countries analyzed, FAOSTAT provides data on the per capita food supply quantity (grams per day), used as a *proxy* of the apparent per capita consumption of these food groups (Table S14 below).

Table S14. Food supply quantity. Grams, per-capita, per day. Source: FAOSTAT (2016)

	Meat	
	Beef	Poultry
Country	g/per capita/per day	
Brazil	35	126
China, mainland	11	37
Ghana	2	15
Indonesia	6	21
Kenya	29	2
Liberia	1	32
Madagascar	17	10
Peru	12	101
Philippines	6	34
Saint Lucia	0	170
Senegal	14	15
South Africa	52	101
Thailand	3	34
Viet Nam	9	26

For the same fourteen countries, drawing upon nutrient data from USDA food composition tables which disseminates reliable data on the composition of foods, beverages, and their ingredients, we compiled an excel sheet with the average nutrient composition of six nutrients (iron, zinc, selenium, calcium, vitamin A and omega-3 fatty acids) per 100 grams of beef and poultry.

[13] https://www.fao.org/faostat/en/#data/SCL
[14] https://fdc.nal.usda.gov/

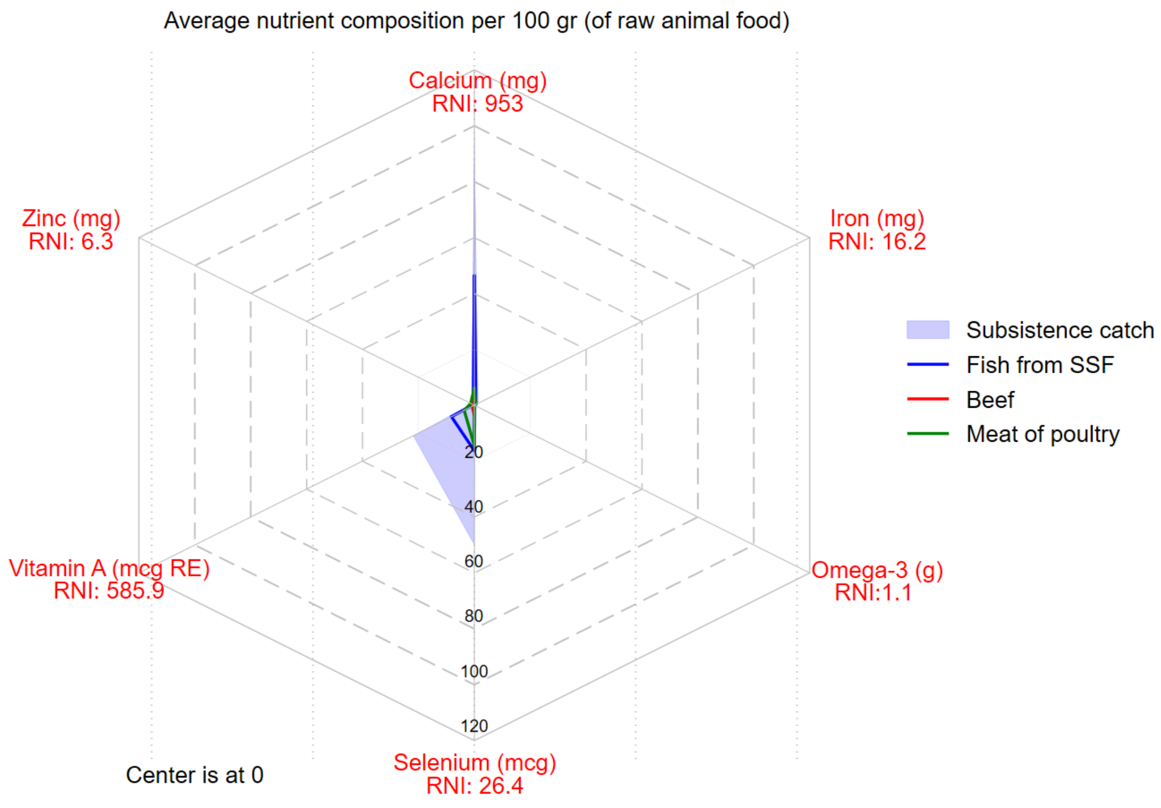

Figure S13. Average food composition across fourteen countries per 100 grams of food groups of six key nutrients. Source: USDA for beef and poultry and IHH data for subsistence catch and fish from SSF.

The daily per-capita apparent nutrient contribution of beef and meat of poultry per 100 grams of food consumed was calculated for six key nutrients as follows:

$$\begin{cases} NC\_calcium_{fg} = intake_{fg} * Nutrient\_calcium_{fg}/100 \\ NC\_selenium_{s} = intake_{fg} * Nutrient\_selenium_{fg}/100 \\ NC\_omega\_3_{s} = intake_{fg} * Nutrient\_omega3_{fg}/100 \\ NC\_zinc_{s} = intake_{fg} * Nutrient\_zinc_{fg}/100 \\ NC\_Vitamin\_a_{s} = intake_{fg} * Nutrient\_Vitamin\_a_{fg}/100 \\ NC\_iron_{s} = intake_{fg} * Nutrient\_iron_{fg}/100 \end{cases}$$

Where index *fg* captures a specific food group. For each of the six nutrients --iron, zinc, selenium, calcium, vitamin A, and omega-3 fatty acids-- the nutrient contribution (“NC_*_{fg}”) was calculated by multiplying the country-specific apparent per capita daily food intake (for the *fg-th* food group) by the average nutrient value ($Nutrient\_*_{fg}$) of the same food group “*fg*”. This calculation allowed generating the absolute per-capita nutrient intake (expressed in milligram per day) from each food group, for six key nutrients.

Finally, to better visualise the nutrient contribution of the food groups, the estimated values for the average apparent per capita consumption of six nutrients from the food groups in the 14 countries were compared to the RNI for the same six nutrients. The RNIs for Calcium, Selenium, Zinc, Iron and Vitamin A were based on the WHO/FAO “Vitamin and mineral requirements in human nutrition[15]”; for Omega-3

[15] https://apps.who.int/iris/bitstream/handle/10665/42716/9241546123.pdf?sequence=1&isAllowed=y

fatty acid, the RNI was based on the "Nutri-Facts[16]" website, which offered scientifically substantiated facts on vitamins, carotenoids and micronutrients, including Omega-3 fatty acid. Since the two sources provided the RNI for different age and sex profiles, the average RNI was calculated by averaging the RNI of different profiles, highlighted in green in Table S11.

This apparent intake of the four food groups is predicted to contribute to an apparent daily intake for the population in the fourteen countries (a total of 2.3 billion people) of their recommended daily intakes of the six key nutrients equivalent to: 11.2 % from poultry and 3.9% from beef The apparent contribution of subsistence catch to the recommended daily intakes of the same six key nutrients for the population dependent upon subsistence activities in SSF, i.e. fishers and their households, (a total of 112.5 million people) is equivalent to 20.1%.

Figure S14. Average contribution of three food groups to the daily RNI for six nutrients, where each bar represents the average percent contribution to the RNI of six nutrients across fourteen countries analyzed. The mean of six nutrients is calculated by averaging the percent contribution to the RNI of the six nutrients.

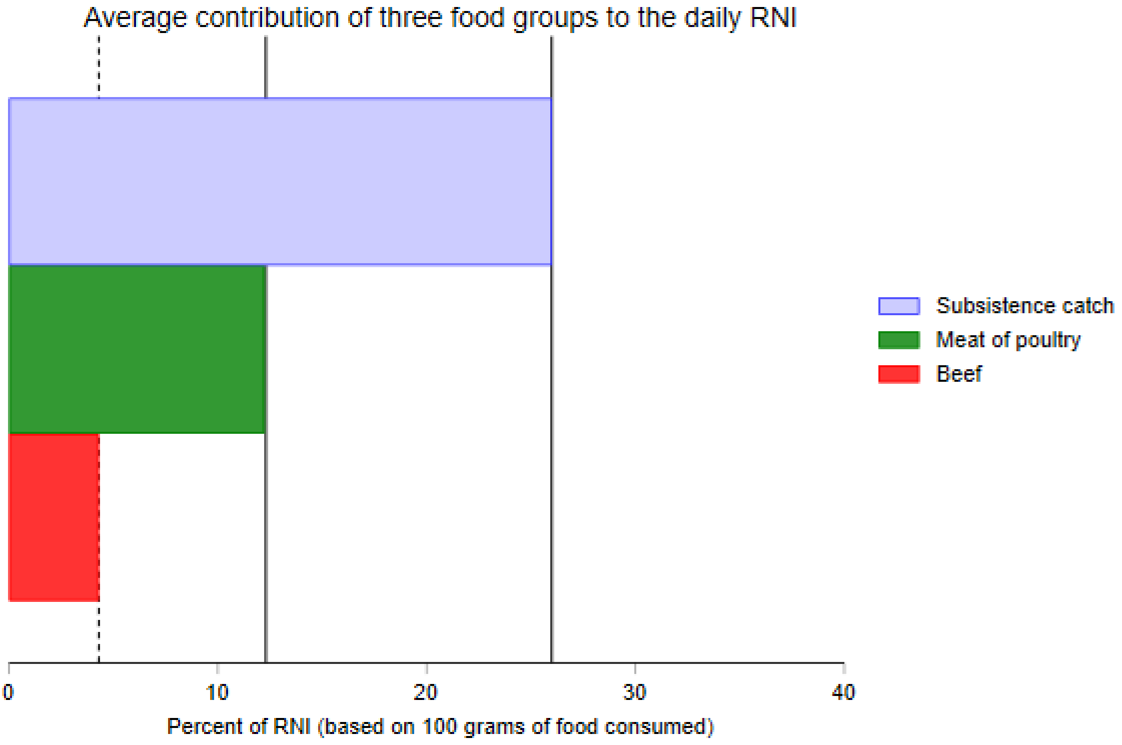

The results from these 14 case studies include important caveats: when not identified by species, the nutrient composition of the estimated subsistence catch was imputed at the level of species group, and the distribution of the subsistence catch and its apparent consumption were assumed rather than measured.

[16] https://www.nutri-facts.org/en_US/nutrients/items/essential-fatty-acids/essential-fatty-acids/intake-recommendations.html

References

Beckett, S. (1995). Stata Technical Bulletin. In *Current* (Issue March).

Ben-Gal, I. (2013). OUTLIER DETECTION. *Data Mining and Knowledge Discovery Handbook: A Complete Guide for Practitioners and Researchers*, 2001–2009.

Crespi. (2004). *Imputation, estimation and prediction using the Key Indicators of the Labour Market (KILM) data set*.

Durrant, G. B. (2005). Imputation methods for handling item-nonresponse in the social sciences: A methodological review. *National Center for Research Methods Working Paper*, *2*, 1–42. http://missingdata.lshtm.ac.uk/preprints/durrantOct05.pdf

FAO. (2021). *COMMITTEE ON FISHERIES: "STATUS UPDATE ON THE GLOBAL STUDY ILLUMINATING HIDDEN HARVESTS: THE CONTRIBUTION OF SMALL-SCALE FISHERIES TO SUSTAINABLE DEVELOPMENT"* (Issue June 2020, pp. 1–3).

FAO, D. U. (2018). *Illuminating Hidden Harvests | FISH CRP*. https://fish.cgiar.org/research-areas/projects/illuminating-hidden-harvests

Hoaglin, D. C. (2016). Regressions are commonly misinterpreted. *Stata Journal*, *16*(1), 5–22. https://doi.org/10.1177/1536867x1601600103

Huang, B. F. F., & Boutros, P. C. (2016). The parameter sensitivity of random forests. *BMC Bioinformatics*, *17*(1), 1–13. https://doi.org/10.1186/s12859-016-1228-x

ICLS, 2013. (2013). ICLS, 2013. *ILO. Resolution I: Resolution Concerning Statistics of Work, Employment and Labour under- Utilization, Adopted by the Nineteenth International Conference of Labour Statisticians, Oct. 2013*, 1–19.

ICSE. (1993). *Resolution concerning the International Classification of Status in Employment (ICSE), adopted by the Fifteenth International Conference of Labour Statisticians*. *36*(3), 1–10.

ILO. (2010). *Trends Econometric Models :*

ILO. (2013). *Decent Work Indicators. Guidelines for producers and users of statistical and legal framework indicators* (Issue December).

ILO. (2017). ILO Labour Forces Estimates and prjections: 1990-2030 Methodological description. *Ilo*, *Vol. 2017*,(No. November,), 1990–2030. https://www.ilo.org/ilostat-files/Documents/LFEP.pdf

ILO. (2018). *ILOSTAT Microdata Processing Quick Guide. Principles and methods underlying the ILO's processing of anonymized household survey microdata*.

Lee, K., & Braithwaite, J. (2020). *High-Resolution Poverty Maps in Sub-Saharan Africa*. http://arxiv.org/abs/2009.00544

Mela, C. F., & Kopalle, P. K. (2002). The impact of collinearity on regression analysis: The asymmetric effect of negative and positive correlations. *Applied Economics*, *34*(6), 667–677. https://doi.org/10.1080/00036840110058482

Oya. (2015). Decent Work Indicators for agriculture and rural areas: Conceptual issues, data collection challenges and possible areas for improvement. *ESS Working Paper*, *October*, 78. www.fao.org/3/a-i5060e.pdf

Roelofs, R., Fridovich-Keil, S., Miller, J., Shankar, V., Hardt, M., Recht, B., & Schmidt, L. (2019). A meta-analysis of overfitting in machine learning. *Advances in Neural Information Processing Systems*, *32*(NeurIPS).

Schaffer, C. (1993). Technical Note: Selecting a Classification Method by Cross-Validation. *Machine Learning*, *13*(1), 135–143. https://doi.org/10.1023/A:1022639714137

Schonlau, M., & Zou, R. Y. (2020). The random forest algorithm for statistical learning. *Stata Journal*, *20*(1), 3–29. https://doi.org/10.1177/1536867X20909688

Smith, H., and Basurto, X. Defining Small-Scale Fisheries and Examining the Role of Science in Shaping Perceptions of Who and What Counts: A Systematic Review. *Frontiers*. doi: https://doi.org/10.3389/fmars.2019.00236

The World Bank. (2017). Fundamentals of Purchasing Power Parities. *International Comparison Program (ICP)*, 2.

UN. (2015). International Standard Industrial Classification of All Economic Activities. In *International Yearbook of Industrial Statistics 2013*. https://doi.org/10.4337/9781781955659.00009

V.J. Hodge J. Austin. (2006). *A Survey of Outlier Detection Methodologies*. http://eprints.whiterose.ac.uk/1783/

World Bank. (2012). Hidden harvest : The global contribution of capture fisheries. *The World Bank. Economic and Sector Work*, *66469*, 92. http://documents.worldbank.org/curated/en/515701468152718292/pdf/664690ESW0P1210120HiddenHarvest0web.pdf

Yeh, I. C., & Lien, C. hui. (2009). The comparisons of data mining techniques for the predictive accuracy of probability of default of credit card clients. *Expert Systems with Applications*, *36*(2 PART 1), 2473–2480. https://doi.org/10.1016/j.eswa.2007.12.020
