# Supplementary material for: Fishing for subsistence constitutes a livelihood safety net for populations dependent on aquatic foods around the world
Source: Nat Food. 2023 Sep 25;4(10):874–85. doi: 10.1038/s43016-023-00844-4 (PMC10589092; doi:10.1038/s43016-023-00844-4)
Supplement: Supplementary file 2 — Reporting Summary [file 43016_2023_844_MOESM2_ESM.pdf]

nature portfolio

Corresponding author(s): John Virdin

Last updated by author(s): Aug 7, 2023

Reporting Summary

Nature Portfolio wishes to improve the reproducibility of the work that we publish. This form provides structure for consistency and transparency in reporting. For further information on Nature Portfolio policies, see our Editorial Policies and the Editorial Policy Checklist.

Statistics

For all statistical analyses, confirm that the following items are present in the figure legend, table legend, main text, or Methods section.

n/a	Confirmed	
☐	☒	The exact sample size (*n*) for each experimental group/condition, given as a discrete number and unit of measurement
☒	☐	A statement on whether measurements were taken from distinct samples or whether the same sample was measured repeatedly
☒	☐	The statistical test(s) used AND whether they are one- or two-sided<br>*Only common tests should be described solely by name; describe more complex techniques in the Methods section.*
☒	☐	A description of all covariates tested
☒	☐	A description of any assumptions or corrections, such as tests of normality and adjustment for multiple comparisons
☐	☒	A full description of the statistical parameters including central tendency (e.g. means) or other basic estimates (e.g. regression coefficient) AND variation (e.g. standard deviation) or associated estimates of uncertainty (e.g. confidence intervals)
☒	☐	For null hypothesis testing, the test statistic (e.g. *F*, *t*, *r*) with confidence intervals, effect sizes, degrees of freedom and *P* value noted<br>*Give P values as exact values whenever suitable.*
☒	☐	For Bayesian analysis, information on the choice of priors and Markov chain Monte Carlo settings
☒	☐	For hierarchical and complex designs, identification of the appropriate level for tests and full reporting of outcomes
☒	☐	Estimates of effect sizes (e.g. Cohen's *d*, Pearson's *r*), indicating how they were calculated

Our web collection on statistics for biologists contains articles on many of the points above.

Software and code

Policy information about availability of computer code

Data collection	Stata 15 and Excel 2019
Data analysis	Stata 15

For manuscripts utilizing custom algorithms or software that are central to the research but not yet described in published literature, software must be made available to editors and reviewers. We strongly encourage code deposition in a community repository (e.g. GitHub). See the Nature Portfolio guidelines for submitting code & software for further information.

Data

Policy information about availability of data

All manuscripts must include a data availability statement. This statement should provide the following information, where applicable:

- Accession codes, unique identifiers, or web links for publicly available datasets
- A description of any restrictions on data availability
- For clinical datasets or third party data, please ensure that the statement adheres to our policy

The results for estimated livelihoods by country and the references for sources are publicly available. The source data from the national household surveys conducted by governments are available from each government's National Statistics Office, which were used with permission for the current study, and are generally publicly available upon request. Data are also available from the authors upon request and with permission of the government's National Statistics Office. The results presented and sources of data (surveys used) are available in the Supplementary Data file.

Human research participants

Policy information about studies involving human research participants and Sex and Gender in Research.

Reporting on sex and gender	Not applicable
Population characteristics	Not applicable
Recruitment	Not applicable
Ethics oversight	No Institutional Review Board (IRB) protocol required.

Note that full information on the approval of the study protocol must also be provided in the manuscript.

Field-specific reporting

Please select the one below that is the best fit for your research. If you are not sure, read the appropriate sections before making your selection.

☐ Life sciences ☐ Behavioural & social sciences ☒ Ecological, evolutionary & environmental sciences

For a reference copy of the document with all sections, see nature.com/documents/nr-reporting-summary-flat.pdf

Ecological, evolutionary & environmental sciences study design

All studies must disclose on these points even when the disclosure is negative.

Study description	Analysis of existing data from 78 standardized national household-based surveys, from 2008 - 2018. These standardized surveys were either: population censuses (PCs), labor force surveys (LFS) or household income and expenditure surveys (HIES) conducted by governments' national statistics agencies. To fill in any gaps in data in surveys and to estimate results for any countries without surveys, we used standard econometric modeling (weighted regression analysis). For the section on nutrition, the subsistence catch volume data was used from country case studies conducted in the global Illuminating Hidden Harvests study, and a predictive modeling approach developed for that study was used to predict the nutrient composition of the catch. Data gaps were filled using the mean nutrient values of species belonging to the same country, same sector or detailed functional group.
Research sample	All countries for which surveys have been conducted and data available were used.
Sampling strategy	There was no sampling procedure.
Data collection	Data was extracted from the survey micro-data and stored in Microsoft Excel for subsequent analysis.
Timing and spatial scale	Data was extracted from national household-based surveys during the period from 2019 – 2020.
Data exclusions	No data were excluded from the analysis
Reproducibility	No experiments were conducted
Randomization	Not applicable because we used all of the data that was available
Blinding	Not applicable because no experiments were conducted.

Did the study involve field work? ☐ Yes ☒ No

Reporting for specific materials, systems and methods

We require information from authors about some types of materials, experimental systems and methods used in many studies. Here, indicate whether each material, system or method listed is relevant to your study. If you are not sure if a list item applies to your research, read the appropriate section before selecting a response.

Materials & experimental systems

n/a	Involved in the study
☒	☐ Antibodies
☒	☐ Eukaryotic cell lines
☒	☐ Palaeontology and archaeology
☒	☐ Animals and other organisms
☒	☐ Clinical data
☒	☐ Dual use research of concern

Methods

n/a	Involved in the study
☒	☐ ChIP-seq
☒	☐ Flow cytometry
☒	☐ MRI-based neuroimaging
